# Bone metastasis and pain research through the dual lens of bibliometrics and bioinformatics: knowledge structure, frontiers, and core pathway analysis (2015–2024)

**DOI:** 10.3389/fmed.2025.1619607

**Published:** 2025-08-12

**Authors:** Linghan Meng, Jingna Tao, Guangda Zheng, Juanxia Ren, Lu Shang, Dongtao Li, Haixiao Liu, Yanju Bao, Baojin Hua

**Affiliations:** ^1^Beijing University of Chinese Medicine, Beijing, China; ^2^Guang’anmen Hospital, China Academy of Chinese Medical Sciences, Beijing, China; ^3^Dongzhimen Hospital, Beijing University of Chinese Medicine, Beijing, China; ^4^Liaoning University of Traditional Chinese Medicine, Shenyang, China

**Keywords:** bone metastasis, cancer-induced bone pain, bibliometrics, bioinformatics, molecular signalling pathways

## Abstract

**Background:**

Cancer-induced bone pain (CIBP) represents a formidable clinical challenge with complex mechanisms, and existing treatments remain unable to effectively control pain in many patients. This study combined bibliometric and bioinformatic approaches to delineate key research trends, principal themes and future directions in the field of bone metastasis and pain research over the past decade (2015–2024).

**Methods:**

We selected 1,822 relevant documents from the Web of Science Core Collection for bibliometric analysis to identify major research characteristics, collaboration networks and emerging trends. Additionally, we employed bioinformatic methods to screen core genes associated with both bone metastasis and cancer pain, and analysed their functions and signalling pathways.

**Results:**

Research output and academic influence demonstrated an upward trajectory, with the United States and China being the countries with the highest publication volumes. Research hotspots are shifting from traditional palliative treatments towards precision therapies, with stereotactic body radiotherapy, minimally invasive ablation techniques and neuropathic pain mechanisms representing major research frontiers. Bioinformatic analysis identified core hub genes such as TP53 and EGFR, and revealed significant enrichment of signalling pathways including PI3K-Akt, MAPK and TNF in the common pathological processes of bone metastasis and pain.

**Conclusion:**

This study, for the first time combining both methodologies, revealed the field’s evolution from traditional treatments towards precision interventions and mechanistic exploration. The molecular targets and signalling pathways we identified provide promising directions for developing novel therapies capable of simultaneously controlling tumour progression and alleviating pain.

## Graphical abstract

Graphical abstract summarizing the dual bibliometric-bioinformatic analysis of research on bone metastasis and pain (2015–2024).
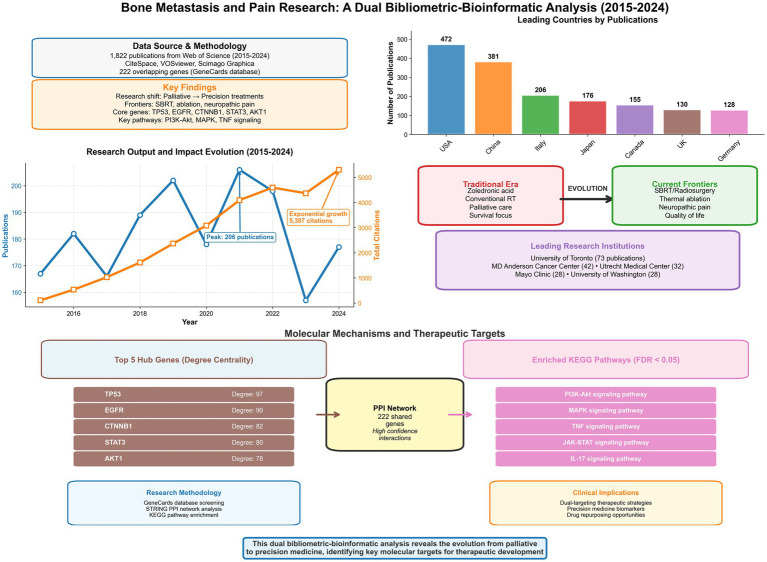

## 1 Introduction

Distant metastasis is one of the critical hallmarks of malignancy, with the skeleton being amongst the most frequently invaded target organs for numerous common cancers (such as breast, prostate and lung) (1–3). Bone metastasis not only signifies disease progression but also precipitates a series of severe complications, with intractable pain being the most predominant and distressing symptom for patients (4, 5). This cancer-induced bone pain (CIBP) is not a singular phenomenon; rather, its underlying mechanisms are intricate and complex, interweaving tumour cell destruction, inflammatory responses, neural damage and remodelling, along with various other pathophysiological processes (1, 6, 7). It casts a persistent shadow, profoundly eroding patients’ quality of life, limiting their mobility, and frequently accompanied by negative psychological states such as anxiety and depression, presenting enormous challenges to patients, their families and indeed the entire healthcare system.

Despite the continuous evolution of therapeutic approaches for CIBP, ranging from conventional analgesics to targeted therapies, inadequate pain control remains widespread in clinical settings, with existing treatments often accompanied by limitations and adverse effects (8, 9). Nevertheless, driven by advances in basic research and clinical imperatives, the past decade (2015–2024) has witnessed an exponential growth in global research on the comorbidity of tumour bone metastasis and pain. Confronted with such rapidly accumulating vast literature, our understanding of the field’s overall knowledge structure, evolutionary trajectory, core research forces and potential research frontiers tends to be fragmented and lagging. Traditional narrative reviews, whilst offering depth, struggle to avoid selection bias and cannot objectively and comprehensively depict the macroscopic landscape and dynamic trends of the entire research domain. Consequently, there is an urgent need for a systematic approach to navigate this complex and rapidly developing field, identifying genuine research hotspots and knowledge gaps that remain insufficiently explored.

To address this challenge, our study introduces bibliometrics, a powerful quantitative analytical tool. Bibliometrics enables objective revelation of knowledge domain development trends, collaboration patterns, research hotspots and frontier dynamics through statistical and visualisation analyses of authors, institutions, countries, keywords and citations in literature from specific fields (10, 11). It functions akin to mapping scientific territories, aiding our navigation through the vast ocean of literature to identify knowledge peaks, pathways and future directions.

However, whilst traditional bibliometric analysis can provide a macroscopic delineation of knowledge maps and developmental trends within research fields, it cannot itself reveal the biological mechanisms underlying these macroscopic phenomena. Bioinformatics analysis, conversely, can uncover core genes and signalling pathways associated with diseases at the molecular level. Therefore, this study innovatively combines these two methodologies to construct an analytical framework spanning from macroscopic research trends to microscopic molecular clues. We aim to identify key evolutionary patterns and frontier hotspots in this field through bibliometrics, whilst employing bioinformatics methods to preliminarily explore the potential common molecular foundations underlying these clinical hotspots, thereby providing a data-driven, multi-layered perspective for understanding the complex associations between bone metastasis and pain, and proposing novel scientific hypotheses for future mechanistic research and target validation.

To systematically present the analytical framework and core findings of this study, we constructed a comprehensive visual summary (graphical abstract). This figure integrates the entire process from data sources and research methods to key bibliometric indicators (such as major contributing countries, institutions and research output trends) as well as core bioinformatics results (including key hub genes and signalling pathways). It intuitively demonstrates how this study, through a dual perspective, reveals the field’s evolution from traditional palliative therapy towards precision medicine, ultimately providing novel insights for clinical practice. In this study, we employed visualisation analysis software such as CiteSpace and VOSviewer, combined with bioinformatics methods, to conduct a comprehensive bibliometric analysis of research papers and reviews concerning cancer-induced bone pain (CIBP) indexed in the Web of Science Core Collection from 2015 to 2024. We systematically depicted the growth curve of research output and the evolution of citation impact in this field over the past decade; identified core research countries, institutions and author groups, and analysed the characteristics of their collaborative networks; determined key journals and knowledge foundations in the field; revealed research hotspots (such as pain mechanisms, therapeutic targets, assessment methods), thematic structures and emerging frontiers through keyword co-occurrence, clustering and burst detection analyses (a method to identify research topics with a sharp increase in attention over a specific period); and further explored core gene targets and signalling pathways associated with CIBP through protein–protein interaction network and KEGG pathway enrichment analyses. Our analysis not only outlines the research panorama of this field but also indicates potential future research directions and therapeutic targets, aiming to provide data-driven insights and valuable references for advancing both basic research and clinical translation in tumour bone metastasis pain.

## 2 Materials and methods

### 2.1 Data collection and search strategy

This research data was collected on 16 April 2025 via the Web of Science Core Collection.^1^ The Web of Science Core Collection (WoSCC) was selected as the data source for this study because it comprehensively indexes high-impact international journals and provides detailed citation data, which is crucial for conducting robust bibliometric analyses such as co-citation and burst analyses. Whilst other databases such as PubMed offer broader coverage in the life sciences field, WoSCC’s structured metadata is particularly well-suited for the software and analytical methods employed in this study. Search parameters were set as follows: databases selected were Science Citation Index Expanded (SCI-EXPANDED) and Social Sciences Citation Index (SSCI); timespan was from 1 January 2015 to 31 December 2024; document types were limited to research articles and reviews; language was restricted to English. The specific search strategy employed topic word combination searches (Table 1), initially yielding 2,047 documents.

**Table 1:** Web of Science Core Collection search strategy.

Set	Results	Search query
#1	13,251	TS = (“bone metast*” OR “skeletal metast*” OR “osseous metast*”)
#2	388,058	TS = (“pain” OR “cancer pain” OR “bone pain” OR “nociception”)
#3	2047	#1 AND #2

**Figure 1:**
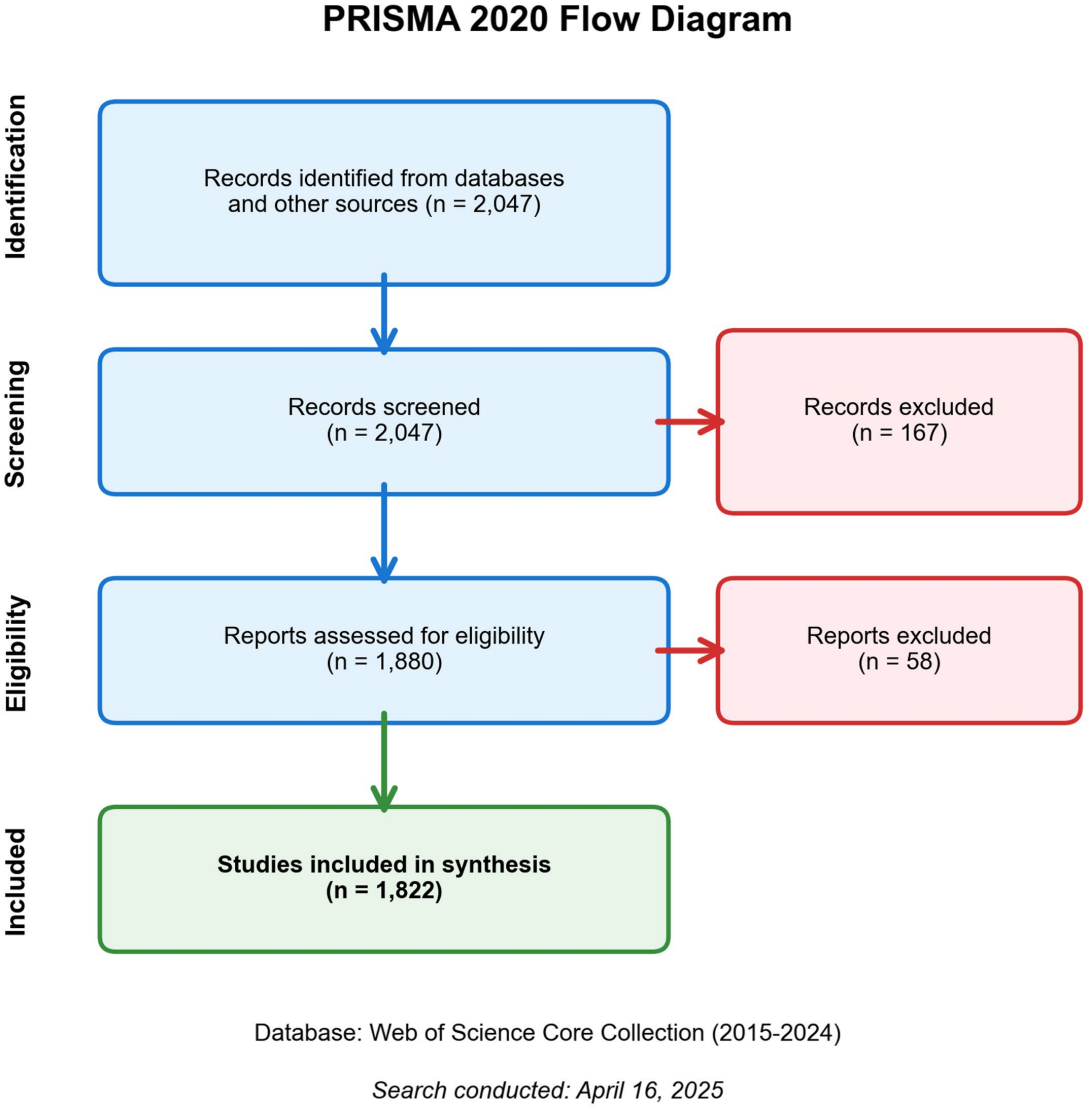
PRISMA 2020 flow diagram of the literature screening and inclusion process.

After secondary screening to exclude conference abstracts, conference papers, editorials, book chapters and other non-target document types, 1,880 documents were retained. Following refinement by English language, 1,822 eligible documents were ultimately included. Data were exported in plain text format and tab-delimited format to accommodate the input requirements of CiteSpace (6.4.R1) and VOSviewer software, respectively. The detailed process of literature screening and selection is illustrated in the PRISMA flow diagram (Figure 1).

### 2.2 Analytical tools

This study employed a multi-tool collaborative analytical framework, including:

(1) Knowledge mapping analysis: CiteSpace (6.4.R1), a software renowned for its powerful capabilities in identifying research frontiers and dynamic evolution, particularly excelling through its unique burst detection algorithm in capturing research themes experiencing rapid increases in attention during specific periods.
(2) Scientometric analysis: VOSviewer (1.6.19), a software distinguished by its exceptional network layout and visualisation capabilities, particularly suited for processing large-scale literature data and generating intuitive and clear networks of author, institutional and country collaborations, as well as keyword co-occurrence networks.
(3) Geographic visualisation: Scimago Graphica (1.0.25), selected for its ability to integrate collaboration network data with world maps, intuitively displaying geographic collaborative relationships and intensity between countries/regions.
(4) Statistical analysis: Microsoft Excel, employed for basic descriptive statistical analysis and chart creation.

This study employed CiteSpace and VOSviewer in combination to achieve complementary advantages: utilising CiteSpace to deeply explore the temporal dynamics and frontier trends of research themes, whilst leveraging VOSviewer to clearly display static collaborative structures and thematic clusters.

#### 2.2.1 Bibliometric parameter settings

CiteSpace analysis parameters were configured as follows: (1) Time slicing: 2015–2024 (annual slices), (2) Threshold selection: g-index (K = 5), (3) Network optimisation: Pathfinder algorithm for pruning slice networks, with secondary pruning after network merging, and (4) Visualisation metrics: Node diameter positively correlated with occurrence frequency, line width reflecting co-occurrence strength.

#### 2.2.2 VOSviewer analysis process

The VOSviewer analysis process included: (1) Data preprocessing: Converting Web of Science export files to UTF-8 encoding, (2) Network construction: Layout optimisation based on LinLog modularity algorithm, and (3) Weight calculation: Node size positively correlated with publication volume (documents) and citation volume (citations).

#### 2.2.3 Scimago Graphica

Scimago Graphica was primarily used to analyse country collaboration networks. Country collaboration tables in GML format obtained from VOSviewer were imported into Scimago Graphica with the following parameter settings: (1) Label selection: country and (2) Cluster selection: string country collaboration network diagrams were created, with country publication volume mapped to node diameter and frequency of collaboration between countries determining line thickness.

#### 2.2.4 Microsoft excel

Microsoft Excel was primarily employed for descriptive statistical analysis, creating pie charts, bar charts, column charts and other visualisations from the various data obtained.

### 2.3 Construction of PPI network and KEGG pathway acquisition for bone metastasis and associated pain

Target genes were retrieved from the GeneCards database^2^ using “Bone metastasis” and “Cancer pain” as keywords, selecting high-scoring genes with relevance scores greater than 20. The GeneCards relevance score is calculated based on the comprehensive association strength between genes and specific keywords across multidimensional biological data (including publications, gene expression, disease associations, etc.). According to the database’s scoring mechanism, scores greater than 10 are considered to indicate significant associations. To further focus on the most core candidate genes and enhance the signal-to-noise ratio of subsequent analyses, this study employed a more stringent threshold (>20) that is commonly adopted in similar bioinformatics literature, ensuring that included genes possessed the strongest associations supported by multi-source evidence. A Venn diagram was created using Wei Sheng Xin—an online bioinformatics analysis and visualisation cloud platform^3^—to identify comorbidity-related genes. The intersection genes were imported into the STRING database,^4^ with “*Homo sapiens*” selected as the organism for searching, using the following settings: minimum required interaction score = high confidence (0.700). According to the official definition of the STRING database, the 0.700 score threshold represents “high confidence” interactions, meaning these interactive relationships are primarily supported by experimentally validated data (such as protein complex purification), co-occurrence in authoritative pathway databases (such as KEGG, Reactome), or evidence repeatedly confirmed through high-throughput experiments. This stringent standard was adopted to construct a high-quality, low false-positive protein–protein interaction network, thereby ensuring the reliability and accuracy of subsequent topological analyses and pathway enrichment analyses. The option to hide disconnected nodes in the network was also selected. The resulting PPI network was exported in TSV format and imported into Cytoscape_v3.7.2 software^5^ for visualisation.

Based on the R 4.4.2 platform,^6^ the clusterProfiler 4.6.2 package was used to conduct KEGG pathway enrichment analysis on the intersection genes. The Benjamini-Hochberg method was applied to correct for multiple hypothesis testing, with significant pathways selected (FDR < 0.05). After excluding human disease-related pathways, the top 15 signalling pathways were selected to construct a “gene-pathway” regulatory network, which was visualised using Cytoscape.

## 3 Results

### 3.1 Analysis of basic characteristics and annual trends of literature

Based on the established search strategy and screening criteria, this study ultimately included 1,822 documents on CIBP research from the Web of Science Core Collection. Analysis of these documents by type revealed that research articles constituted the predominant form of literature, totalling 1,400 documents (76.84%), followed by reviews, numbering 422 documents (23.16%). This indicates that research in this field is primarily characterised by original exploratory studies, whilst also accumulating a considerable number of summative review documents.

**Figure 2:**
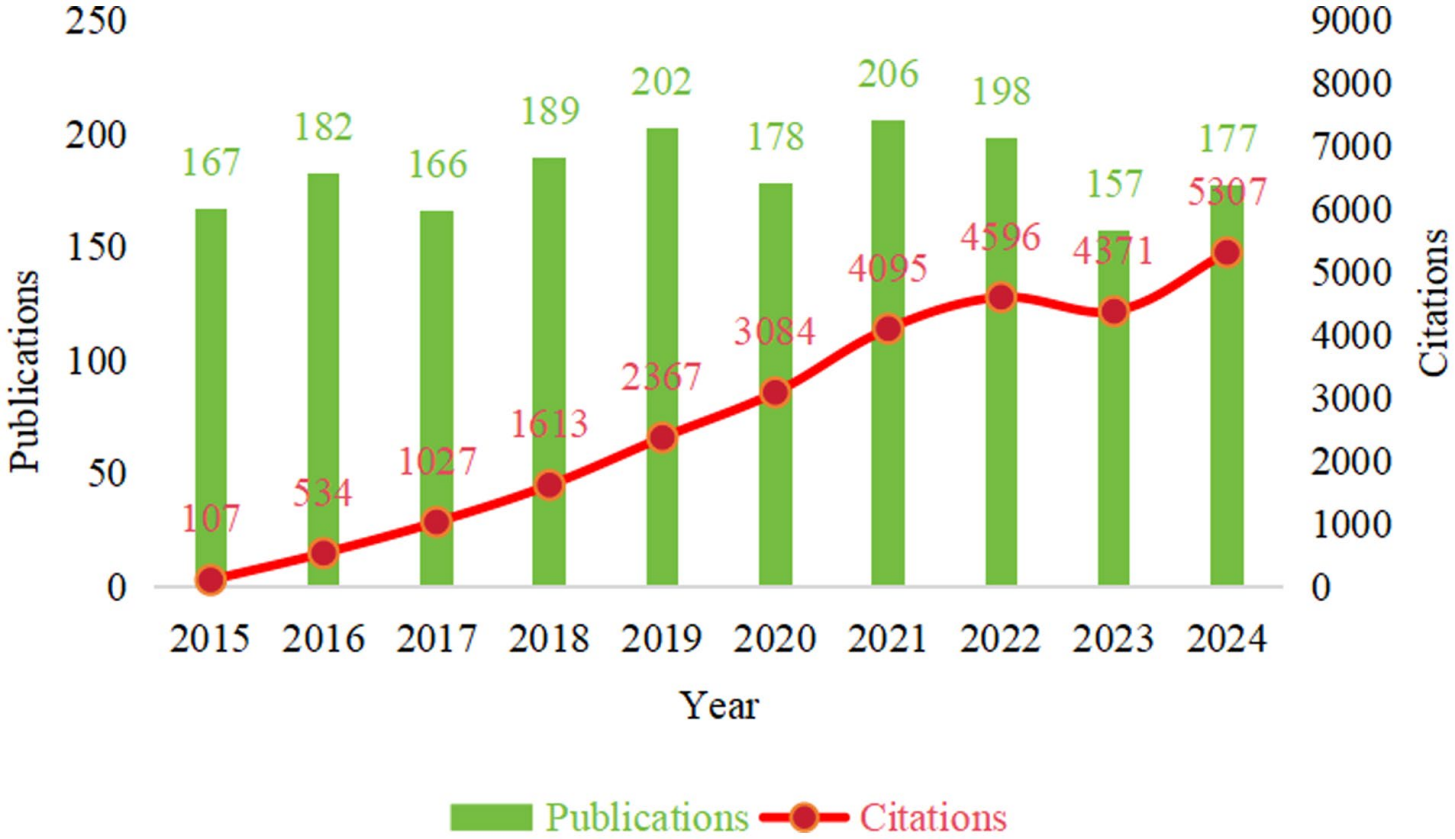
Annual publication volume and total citation frequency trends (2015–2024).

To understand the developmental trajectory of research in this field, we analysed the annual publication volume and citation frequency trends between 2015 and 2024 (Figure 2). As shown in Figure 2, the annual publication volume exhibited an overall fluctuating upward trend. Starting with 167 publications in 2015, and after experiencing minor fluctuations, the volume reached two relative peaks in 2019 (202 publications) and 2021 (206 publications), subsequently declining somewhat in 2023 (157 publications), with 177 publications in 2024. This indicates that research in the field of CIBP has remained consistently active over the past decade, and despite annual output fluctuations, has generally maintained a high level.

Concurrently, the annual total citation frequency demonstrated a continuous and significant growth trend (Figure 2, red curve), rapidly climbing from 107 citations in 2015 to 5,307 citations in 2024. This rapid growth in citation frequency strongly confirms the expanding academic influence of research in this field, with relevant research outcomes receiving widespread attention and recognition from the academic community.

### 3.2 Analysis of countries/regions

Statistical results revealed that a total of 79 countries/regions worldwide participated in research on CIBP. The top 10 countries/regions by publication volume and their bibliometric indicators are shown in Table 2.

**Table 2:** Top 10 countries/regions by publication volume and their bibliometric indicators (2015–2024).

Country/region	Documents	Citations	Total link strength
USA	472	11,197	455
China	381	3,743	85
Italy	206	4,590	237
Japan	176	1,992	85
Canada	155	4,722	269
United Kingdom	130	4,783	338
Germany	128	3,790	241
France	116	3,300	186
Netherlands	89	1,882	157
Switzerland	68	2,277	200

**Figure 3:**
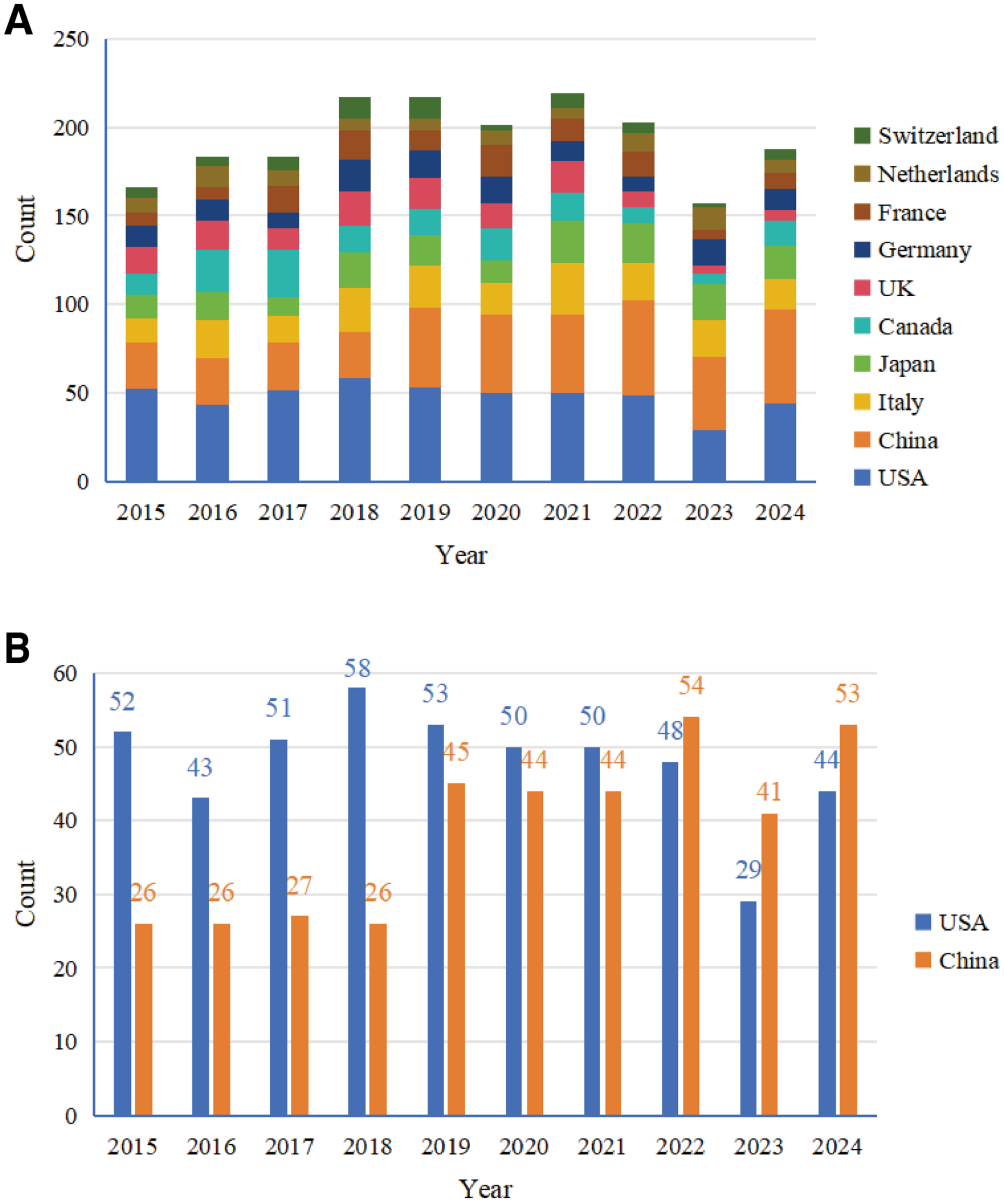
Annual publication trends of major countries (2015–2024). **(A)** Stacked chart of annual publication volumes for the top 10 countries. **(B)** Comparison of annual publication volumes between China and the United States.

Amongst these, the United States led by a considerable margin with 472 publications, accounting for 25.9% of the total literature volume (1,822 documents), whilst its total citation frequency (11,197) also far exceeded that of other countries/regions, demonstrating its absolute leading position and strong international influence in this field. China ranked second with 381 publications (20.9%) and 3,743 total citations. These were followed by Italy (206 publications, 11.3%), Japan (176 publications, 9.7%), Canada (155 publications, 8.6%), the United Kingdom (130 publications, 7.1%) and Germany (128 publications, 7.0%). In terms of Total Link Strength (a metric quantifying the total collaborative strength of a node within the network), the United States (455), the United Kingdom (338) and Canada (269) demonstrated the most active international collaboration. The annual publication trends of the top 10 countries/regions by publication volume are shown in Figure 3A. It is evident that these major countries/regions contributed the vast majority of literature output in this field.

The comparison of annual publication volumes between the United States and China (Figure 3B) shows that during the early research period (2015–2018), the US publication volume was significantly higher than China’s. From 2019 onwards, China’s annual publication volume increased substantially (45 publications in 2019, 44 in 2020, 44 in 2021), and in 2022 (54 publications) slightly exceeded the United States (48 publications) for the first time. Although the US publication volume declined somewhat in 2023 (29 publications), it maintained a high level in 2024 (44 publications), whilst China’s publication volume also remained high during the same period (53 publications), with both countries jointly leading research development in this field.

**Figure 4:**
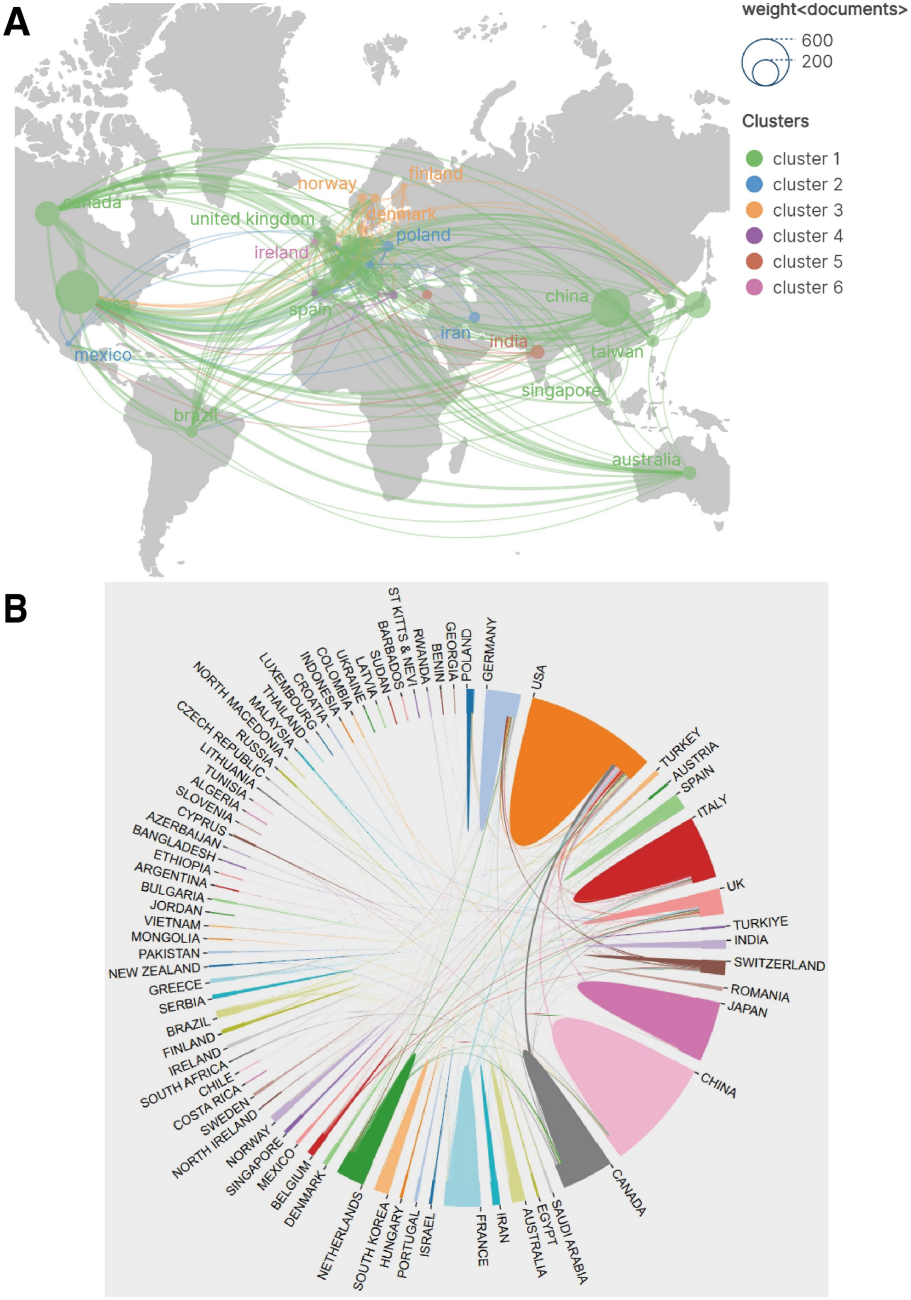
Research collaboration networks between countries/regions. **(A)** Geographic visualisation of collaboration networks, where node size represents publication volume and lines represent collaborative relationships. **(B)** Chord diagram of collaborative relationships, displaying collaboration flow directions and intensity between major countries.

Collaboration networks between countries/regions were visualised through geographic visualisation (Figure 4A) and chord diagrams (Figure 4B).

As shown in Figure 4A, node size represents publication volume and lines on the map represent collaborative relationships. The United States not only had the largest publication volume but also the most extensive collaboration network, maintaining collaborative connections with numerous countries/regions globally, positioning it as the absolute core of the network. Although China ranked second in publication volume, its breadth and intensity of international collaboration (Total Link Strength of 85) still lagged behind Western countries/regions such as the United States, the United Kingdom and Canada, with relatively more concentrated collaborative partners. European countries/regions (such as the United Kingdom, Germany, Italy, France, Switzerland and the Netherlands) formed a relatively dense collaboration network amongst themselves. The chord diagram (Figure 4B) more intuitively displayed the collaboration flow between countries/regions, clearly showing substantial bidirectional or unidirectional collaboration between the United States and Canada, the United Kingdom, China, Italy, Germany and other countries.

### 3.3 Analysis of institutions

**Figure 5:**
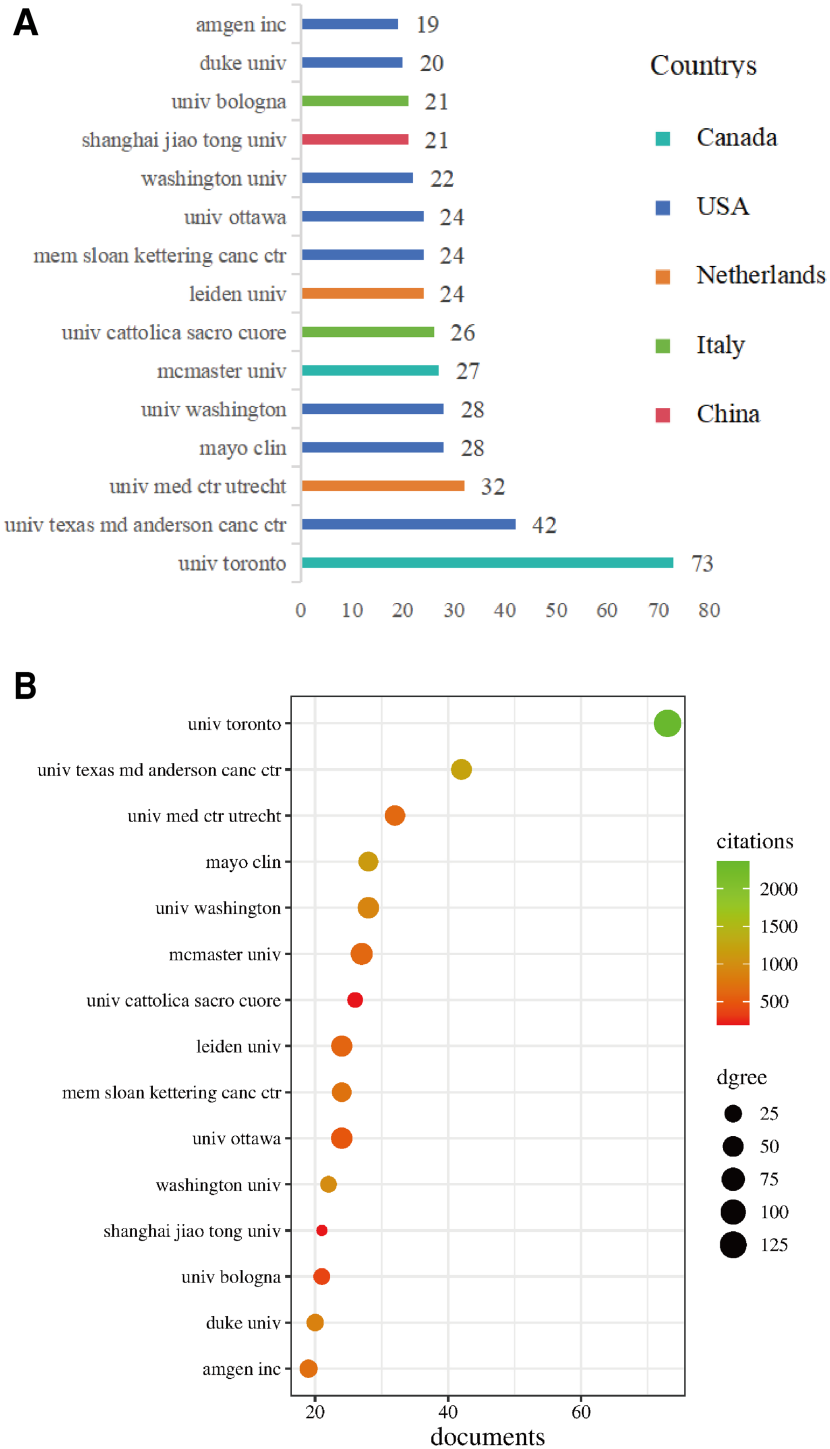
Analysis of major research institutions. **(A)** Distribution of the top 15 institutions by publication volume and their respective countries. **(B)** Bubble chart showing the relationship between publication volume (X-axis), total citation frequency (colour) and collaboration intensity (TLS, node size) of major institutions.

This study identified 65 research institutions worldwide that contributed to research in the field of CIBP. Statistical analysis of publication volume for each institution was conducted, with the distribution of the top 15 institutions by publication volume and their respective countries shown in Figure 5A, and detailed data presented in Table 3.

**Table 3:** Top 15 research institutions by publication volume and their bibliometric indicators.

Organization	Documents	Citations	Total link strength	Country
Univ Toronto	73	2,361	133	Canada
Univ Texas MD Anderson Canc Ctr	42	1,215	49	USA
Univ Med Ctr Utrecht	32	639	47	Netherlands
Mayo Clin	28	1,134	42	USA
Univ Washington	28	895	56	USA
Mcmaster Univ	27	629	61	Canada
Univ Cattolica Sacro Cuore	26	190	15	Italy
Leiden Univ	24	594	53	Netherlands
Mem Sloan Kettering Canc Ctr	24	720	40	USA
Univ Ottawa	24	486	55	USA
Washington Univ	22	974	20	USA
Shanghai Jiao Tong Univ	21	196	5	China
Univ Bologna	21	364	20	Italy
Duke Univ	20	855	24	USA
Amgen Inc.	19	682	29	USA

Results showed that the University of Toronto (Canada) led by a considerable margin with 73 publications, ranking first. This was followed by the University of Texas MD Anderson Cancer Center (USA, 42 publications), University Medical Center Utrecht (Netherlands, 32 publications), Mayo Clinic (USA, 28 publications) and the University of Washington (USA, 28 publications). The top-ranked high-productivity institutions were primarily concentrated in the United States, Canada, the Netherlands and Italy.

In terms of academic influence (Table 3), the University of Toronto also had the highest total citation frequency, reaching 2,361 citations. The University of Texas MD Anderson Cancer Center (1,215 citations), Mayo Clinic (1,134 citations), University of Washington (974 citations) and the University of Sheffield (United Kingdom, 1,096 citations, despite having only 17 publications) also demonstrated outstanding citation frequencies. The institutional influence bubble chart (Figure 5B) intuitively displayed the relationship between publication volume (horizontal axis), total citation frequency (colour depth/warmth) and degree of collaboration (node size, representing Total Link Strength) for major institutions. This chart clearly showed that the University of Toronto held an absolute leading position in terms of publication volume, citation frequency and degree of collaboration. Institutions such as the University of Texas MD Anderson Cancer Center, Mayo Clinic and the University of Washington demonstrated balanced performance in high output and high influence.

**Figure 6:**
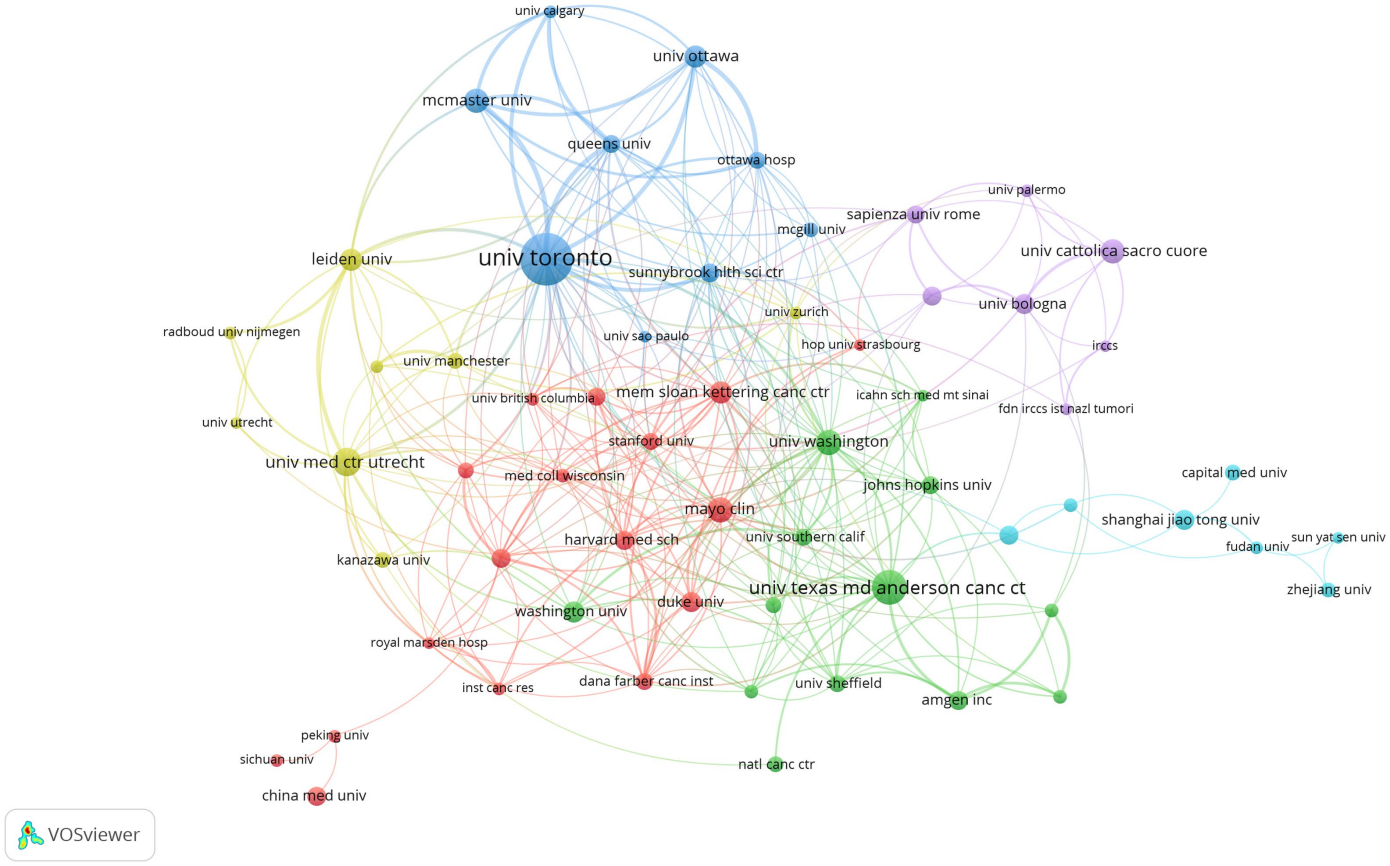
Research institution collaboration network.

To reveal collaboration patterns between institutions, we constructed an institutional collaboration network diagram (Figure 6).

In this network, node size represents an institution’s publication volume, and line thickness represents collaboration intensity. The network exhibited distinct cluster characteristics, forming several major collaborative groups:

Blue cluster: Primarily centred around Canadian institutions such as the University of Toronto, McMaster University, Queen’s University and the University of Ottawa, with close internal connections.

Green cluster: Mainly comprised of top US cancer research institutions, such as the University of Texas MD Anderson Cancer Center, Memorial Sloan Kettering Cancer Center, Dana-Farber Cancer Institute and Amgen Inc., demonstrating the United States’ formidable strength and collaborative network in cancer research.

Red cluster: Included renowned US institutions such as Harvard Medical School, Stanford University, Washington University and Mayo Clinic.

Yellow cluster: Predominantly European institutions, particularly Leiden University and University Medical Center Utrecht in the Netherlands.

Purple cluster: Mainly comprised Italian institutions, such as Sapienza University of Rome, University of Bologna and Catholic University of the Sacred Heart.

Cyan cluster: Primarily composed of Chinese institutions, such as Shanghai Jiao Tong University, Fudan University, Sun Yat-sen University and Capital Medical University.

In terms of Total Link Strength (TLS) (Table 3), the University of Toronto (TLS = 133) demonstrated the most extensive collaboration, followed by McMaster University (TLS = 61), Queen’s University (TLS = 63), University of Ottawa (TLS = 55) and Leiden University (TLS = 53). This indicates that whilst transnational collaboration exists (as shown by connections between clusters of different colours in the figure), collaboration tends to be more concentrated and closer within the same country or region (such as within Canada, between US cancer centres, amongst certain European countries) and between institutions in specific research fields.

### 3.4 Analysis of authors

#### 3.4.1 Analysis of publishing authors

**Figure 7:**
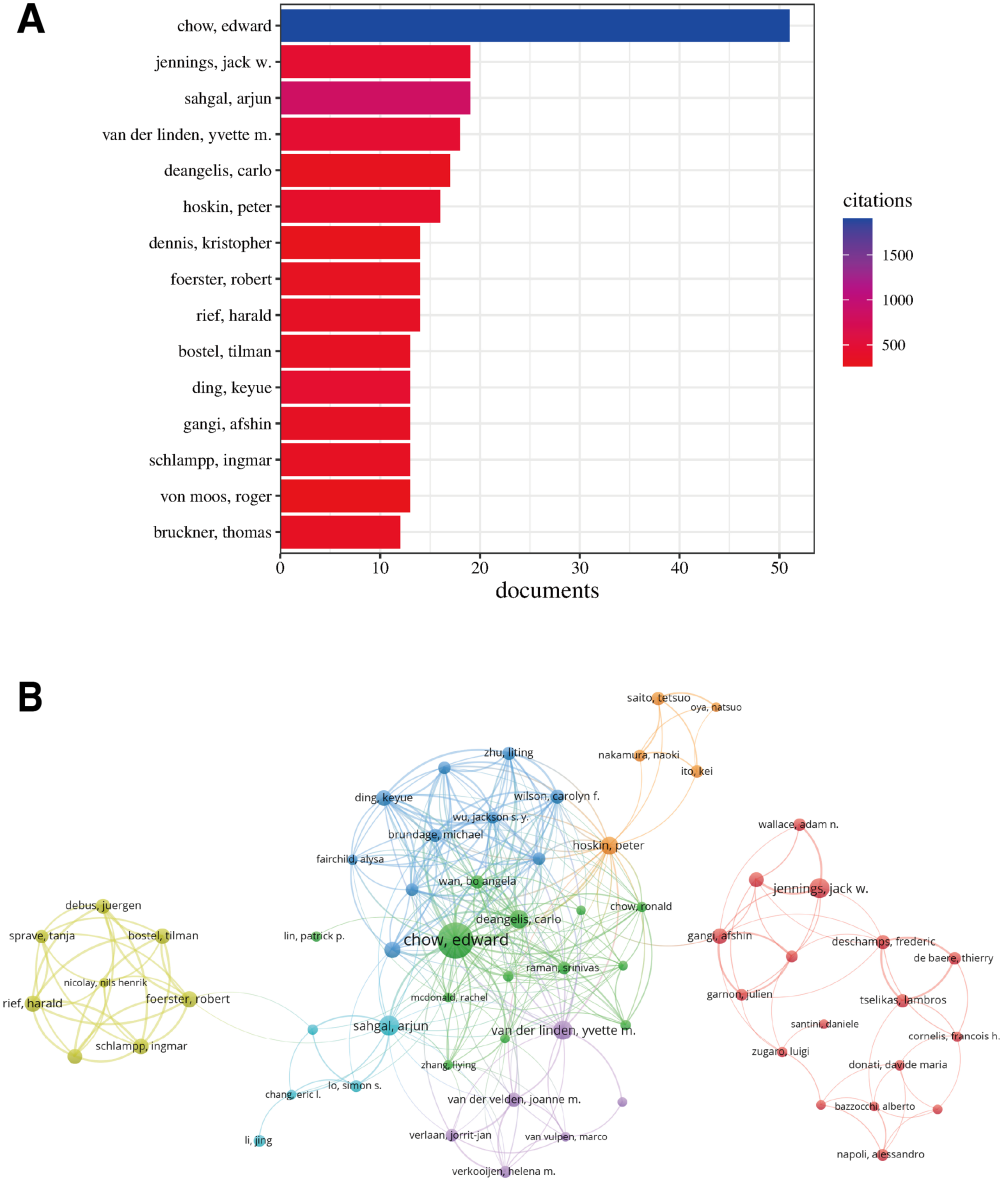
Analysis of highly productive authors and collaboration networks. **(A)** Top 15 authors by publication volume. **(B)** Author collaboration network, where node size represents publication volume, lines represent collaboration strength, and colours represent collaboration clusters.

Statistical analysis of authors’ publication volumes was conducted, with Table 4 and Figure 7A displaying the top 10 and top 15 authors by publication volume, respectively.

**Table 4:** Top 10 authors by publication volume and their bibliometric indicators.

Author	Documents	Citations	Total link strength	Country
Chow, Edward	51	1,904	159	Canada
Jennings, Jack W.	19	444	29	USA
Sahgal, Arjun	19	839	29	Canada
van der Linden, Yvette M.	18	452	54	Netherlands
DeAngelis, Carlo	17	296	80	Canada
Hoskin, Peter	16	401	64	UK
Dennis, Kristopher	14	276	55	Canada
Foerster, Robert	14	324	67	Germany
Rief, Harald	14	343	71	Germany
Bostel, Tilman	13	338	70	Germany

Edward Chow from Canada ranked first with 51 publications, far exceeding other authors, demonstrating his outstanding contribution to this field. Arjun Sahgal, also from Canada, and Jack W. Jennings from the United States jointly ranked second, each with 19 publications. Yvette M. van der Linden from the Netherlands (18 publications) and Carlo DeAngelis from Canada (17 publications) were also amongst the highly productive authors. Overall, highly productive authors primarily came from Canada, the United States, Germany and the Netherlands.

The author collaboration network (Figure 7B) intuitively displayed collaborative relationships between authors. The network exhibited several distinct collaboration clusters (different coloured regions in the figure), indicating the existence of multiple relatively independent research teams in this field. A cluster centred around Canadian authors Edward Chow, Arjun Sahgal and Carlo DeAngelis (green region in the figure) was the largest, with dense internal connections and close collaborative relationships. Another significant cluster primarily comprised German authors including Tilman Bostel, Harald Rief, Ingmar Schlampp, Robert Foerster and Juergen Debus (yellow region in the figure), similarly demonstrating close internal collaboration. Additionally, there were relatively smaller clusters centred around Jack W. Jennings (USA), Afshin Gangi (France) and Frederic Deschamps (France) (red region in the figure). Overall, whilst there were connections between different clusters (representing cross-team or transnational collaboration), most close collaborations occurred between scholars within the same research team or in geographical proximity.

#### 3.4.2 Analysis of cited authors

**Figure 8:**
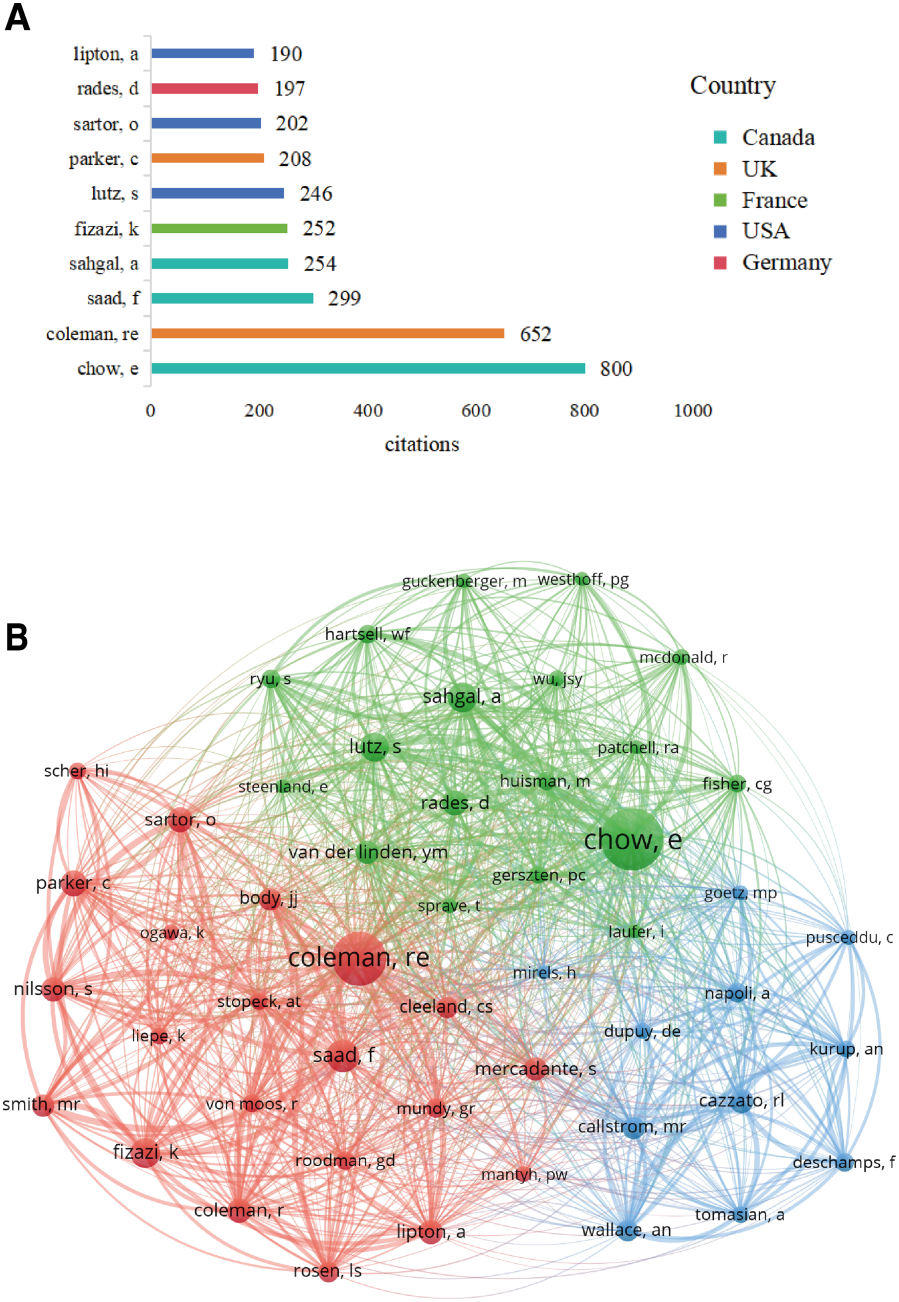
Analysis of highly cited authors and co-citation networks. **(A)** Top 10 authors by citation frequency and their country distribution. **(B)** Author co-citation network, where node size represents citation frequency, lines represent co-citation strength, and colours represent major research clusters.

Author co-citation analysis aimed to identify scholars with high academic influence in this field. Table 5 and Figure 8A list the top 10 scholars by citation frequency and their primary country distribution.

**Table 5:** Top 10 authors by citation frequency and their bibliometric indicators.

Author	Citations	Total link strength	Country
Chow, E	800	5,252	Canada
Coleman, RE	652	4,612	UK
Saad, F	299	2,982	Canada
Sahgal, A	254	2,273	Canada
Fizazi, K	252	2,500	France
Lutz, S	246	2,118	USA
Parker, C	208	1,909	UK
Sartor, O	202	1,918	USA
Rades, D	197	1,647	Germany
Lipton, A	190	1,831	USA

E. Chow from Canada was not only the author with the highest publication volume but also the scholar with the highest citation frequency, reaching 800 citations. RE. Coleman from the UK ranked second with 652 citations. Other highly cited scholars included F. Saad (299 citations) and A. Sahgal (254 citations) from Canada, K. Fizazi (252 citations) from France, S. Lutz (246 citations) from the United States, C. Parker (208 citations) from the UK, O. Sartor (202 citations) from the United States, D. Rades (197 citations) from Germany, and A. Lipton (190 citations) from the United States. These highly cited scholars represented the core strength and significant knowledge contributors in this field of research.

The author co-citation network (Figure 8B) displayed the knowledge association structure amongst these highly influential scholars. The node size reflected the frequency with which an author was cited, with E. Chow and RE. Coleman’s nodes significantly larger than others, positioning them as the core of the network. The network exhibited three main clusters (different colours in the figure):

Cluster 1 (green): Centred around scholars such as E. Chow, A. Sahgal, S. Lutz and D. Rades. Considering these scholars’ research backgrounds, this cluster likely primarily represented research directions in radiotherapy (particularly stereotactic body radiotherapy, SBRT) for bone metastases and related pain management.

Cluster 2 (red): Centred around scholars such as RE. Coleman, F. Saad, K. Fizazi, C. Parker, O. Sartor, A. Lipton and MR. Smith. Scholars in this cluster were closely associated with anti-bone resorption drugs (such as bisphosphonates and denosumab), endocrine therapy and systemic treatment of prostate cancer bone metastases.

Cluster 3 (blue): Included scholars such as RL. Cazzato, MR. Callstrom, F. Deschamps, Afshin Gangi, A. Napoli and A. Tomasian. This cluster might be more associated with interventional treatment of bone metastases (such as ablation and bone cement augmentation), imaging diagnosis and guidance.

These clusters clearly reflected different research schools and important knowledge subdomains within the field of CIBP, revealing the intrinsic connections between scholars based on research content.

### 3.5 Analysis of journals

#### 3.5.1 Analysis of publishing journals

The 1,822 documents included in this study were widely distributed across 65 journals. Table 6 lists the top 10 journals by publication volume and their related indicators.

**Table 6:** Top 10 journals by publication volume and their related indicators.

Source	Documents	Citations	Total link strength	IF	JCR quartile
Medicine	59	276	33	1.4	Q3
Frontiers in Oncology	44	376	73	3.5	Q2
Cancers	37	398	122	4.5	Q2
Journal of Bone Oncology	36	435	107	3.1	Q3
Supportive Care in Cancer	25	386	72	2.8	Q2
Radiotherapy and Oncology	22	786	212	4.9	Q2
International Journal of Radiation Oncology Biology Physics	22	409	158	6.4	Q1
Cardiovascular and Interventional Radiology	17	284	106	2.8	Q3
Practical Radiation Oncology	16	451	109	3.4	Q3
International Journal of Molecular Sciences	12	345	18	4.9	Q1
Journal of Vascular and Interventional Radiology	12	262	95	2.6	Q3
International Journal of Hyperthermia	12	256	63	3.0	Q3
European Journal of Nuclear Medicine and Molecular Imaging	9	343	28	8.6	Q1
Journal of Neurosurgery-Spine	8	305	47	2.9	Q3
European Urology	7	404	18	25.3	Q1

Among these, “Medicine” was the journal that published the most literature in this field, with a total of 59 publications, followed by “Frontiers in Oncology” (44 publications), “Cancers” (37 publications), “Journal of Bone Oncology” (36 publications) and “Annals of Palliative Medicine” (36 publications).

**Figure 9:**
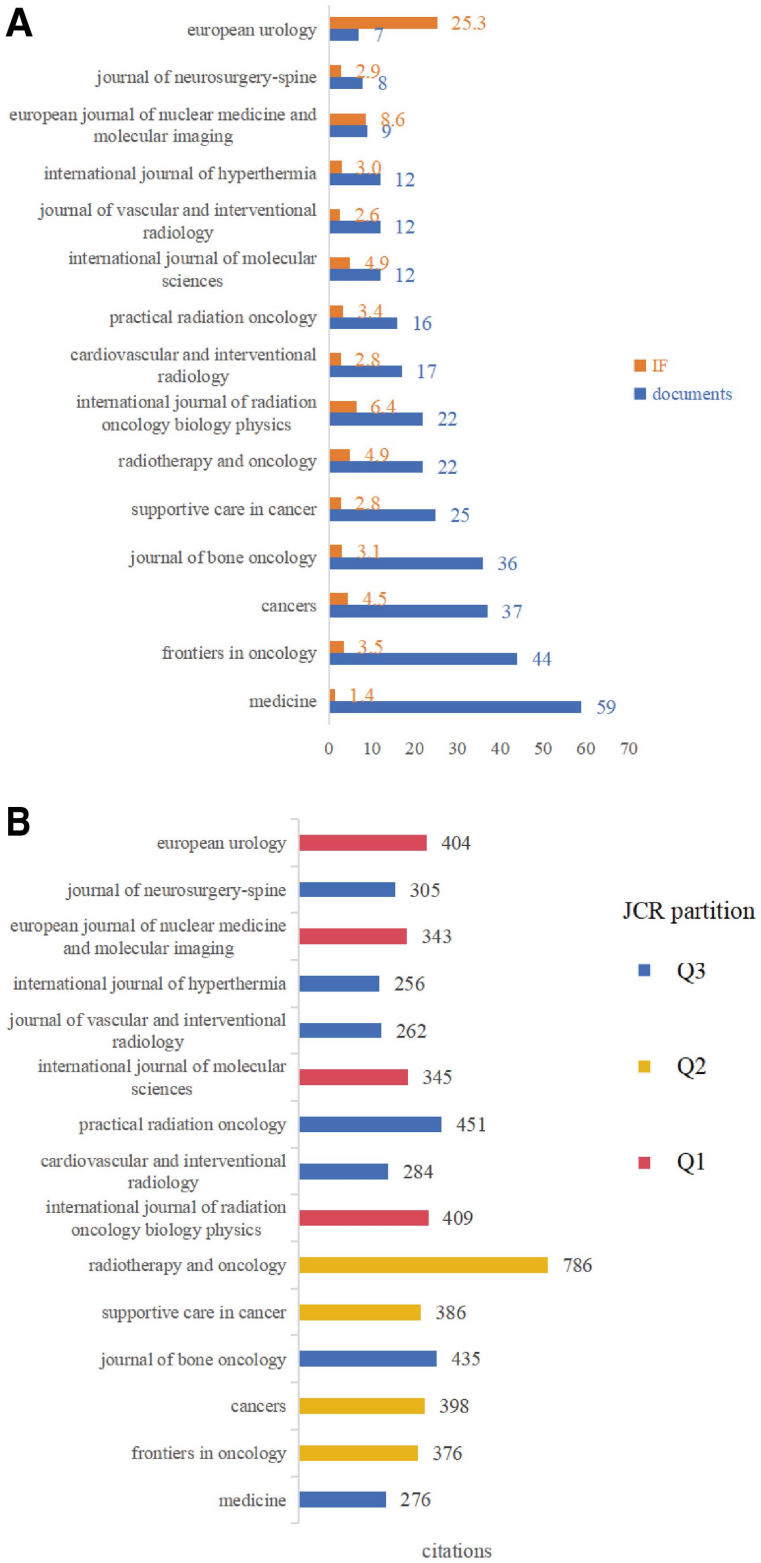
Analysis of characteristics of major publishing journals. **(A)** Relationship between publication volume and impact factor (IF) for the top 15 journals by publication volume. **(B)** Relationship between total citation count in this dataset and JCR quartile for the top 15 journals by publication volume.

To understand the academic influence of these major publishing journals, we analysed their impact factors (IF) and JCR quartiles. Figure 9A shows the relationship between publication volume and impact factor for the top 15 journals by publication volume.

It can be observed that some high-volume journals, such as “Medicine” (IF = 1.4), had relatively low impact factors. Conversely, journals such as “European Urology” (7 publications, IF = 25.3), “International Journal of Radiation Oncology Biology Physics” (22 publications, IF = 6.4) and “European Journal of Nuclear Medicine and Molecular Imaging” (9 publications, IF = 8.6), despite having relatively fewer publications, had very high impact factors, indicating that high-quality research outcomes were also distributed across these top-tier journals.

Figure 9B displays the relationship between total citation count (referring to the number of times literature in this field published in these journals was cited) and JCR quartile for the top 15 journals by publication volume. Results showed that “Radiotherapy and Oncology” (786 citations), “Practical Radiation Oncology” (451 citations) and “Journal of Bone Oncology” (435 citations) were the most cited journals. Regarding JCR quartiles, Q1 journals included “International Journal of Radiation Oncology Biology Physics,” “International Journal of Molecular Sciences,” “European Journal of Nuclear Medicine and Molecular Imaging” and “European Urology”; Q2 journals included “Frontiers in Oncology,” “Cancers,” “Supportive Care in Cancer” and “Radiotherapy and Oncology.” This indicated that research outcomes in this field were published across multiple levels and types of journals, from comprehensive oncology journals to specialty journals (such as bone oncology, radiotherapy, interventional radiology and palliative medicine) as well as high-impact journals.

**Figure 10:**
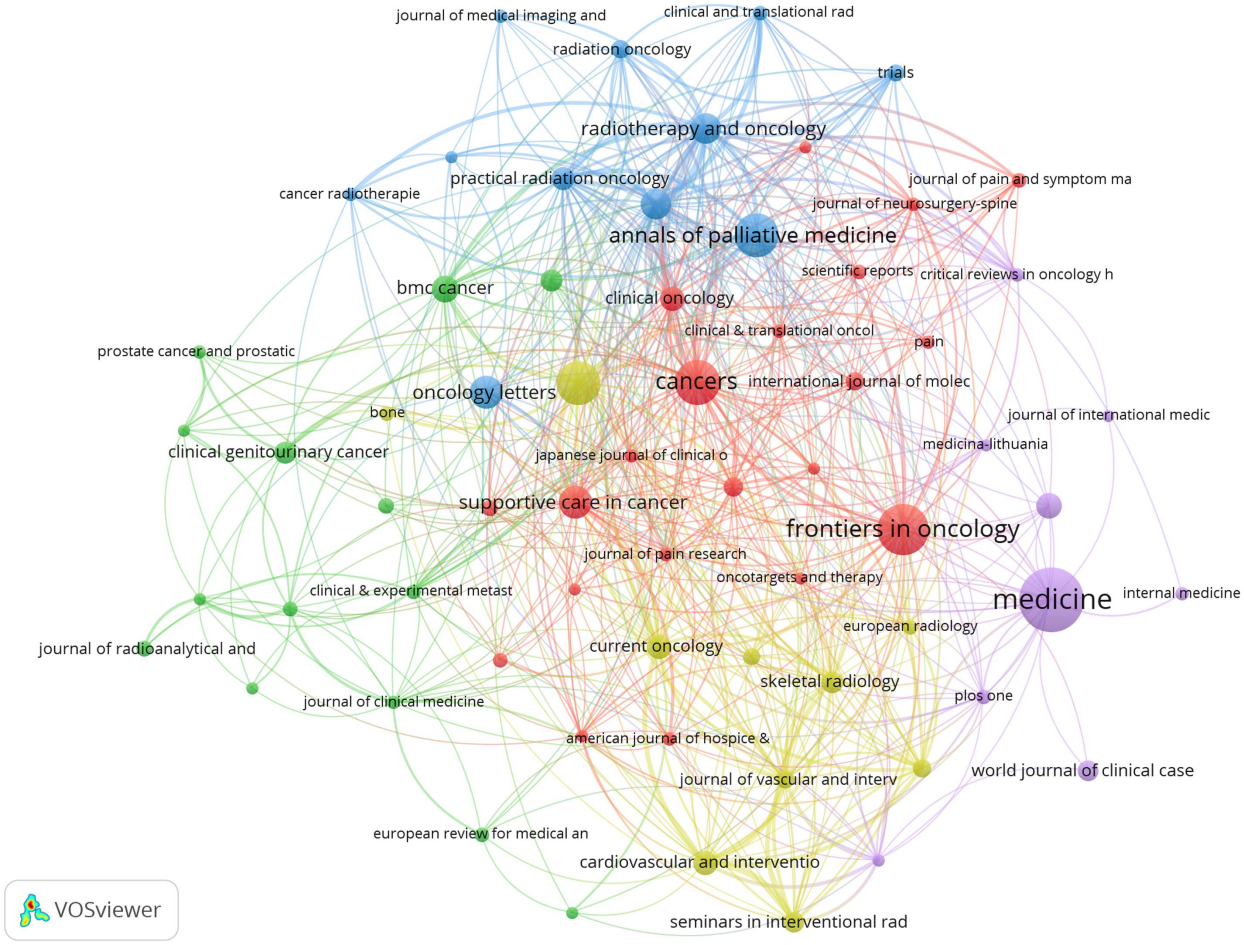
Journal co-occurrence network.

The journal co-occurrence network (Figure 10) revealed thematic connections between journals publishing research in this field.

Several main clusters formed within the network: the red cluster primarily comprised oncology and pain research journals such as “Cancers,” “Frontiers in Oncology,” “Pain” and “Journal of Pain Research”; the blue cluster was mainly composed of radiation oncology journals such as “Radiotherapy and Oncology,” “Practical Radiation Oncology” and “Clinical and Translational Radiation Oncology”; the yellow cluster included “Oncology Letters,” “Supportive Care in Cancer” and “Journal of Vascular and Interventional Radiology”; whilst the green cluster featured “BMC Cancer” and “Clinical & Experimental Metastasis.” This further illustrated the multidisciplinary cross-cutting nature of research in this field, involving multiple disciplinary directions including oncology, pain science, radiation therapy, interventional radiology and palliative medicine.

#### 3.5.2 Analysis of cited journals

Journal co-citation analysis was used to identify the knowledge foundation and core sources of influence for research in this field. Table 7 lists the top 10 journals by citation frequency in this study’s literature dataset.

**Table 7:** Top 10 journals by citation frequency and their related indicators.

Source	Citations	Total link strength	IF	JCR Quartile
J Clin Oncol	2,640	81,537	42.1	Q1
Int J Radiat Oncol	2,402	60,445	6.4	Q1
Radiother Oncol	1,277	35,763	4.9	Q2
New Engl J Med	1,156	35,383	96.3	Q1
Cancer-Am Cancer Soc	1,118	34,485	6.1	Q1
Lancet Oncol	1,107	37,161	41.6	Q1
J Nucl Med	943	25,621	9.1	Q1
Ann Oncol	936	29,150	56.7	Q1
Clin Cancer Res	905	32,718	10.4	Q1
Pain	789	16,777	5.9	Q1

Results showed that “Journal of Clinical Oncology” (J Clin Oncol) was the most cited journal, with 2,640 citations, demonstrating its authoritative position in this field. This was followed by “International Journal of Radiation Oncology Biology Physics” (Int J Radiat Oncol, 2,402 citations) and “Radiotherapy and Oncology” (Radiother Oncol, 1,277 citations), reflecting the importance of radiotherapy in research in this field. Other highly cited journals included top general medical journals such as “New England Journal of Medicine” (1,156 citations) and “Lancet Oncology” (1,107 citations), as well as important oncology journals such as “Cancer-American Cancer Society” (1,118 citations), “Annals of Oncology” (936 citations) and “Clinical Cancer Research” (905 citations).

**Figure 11:**
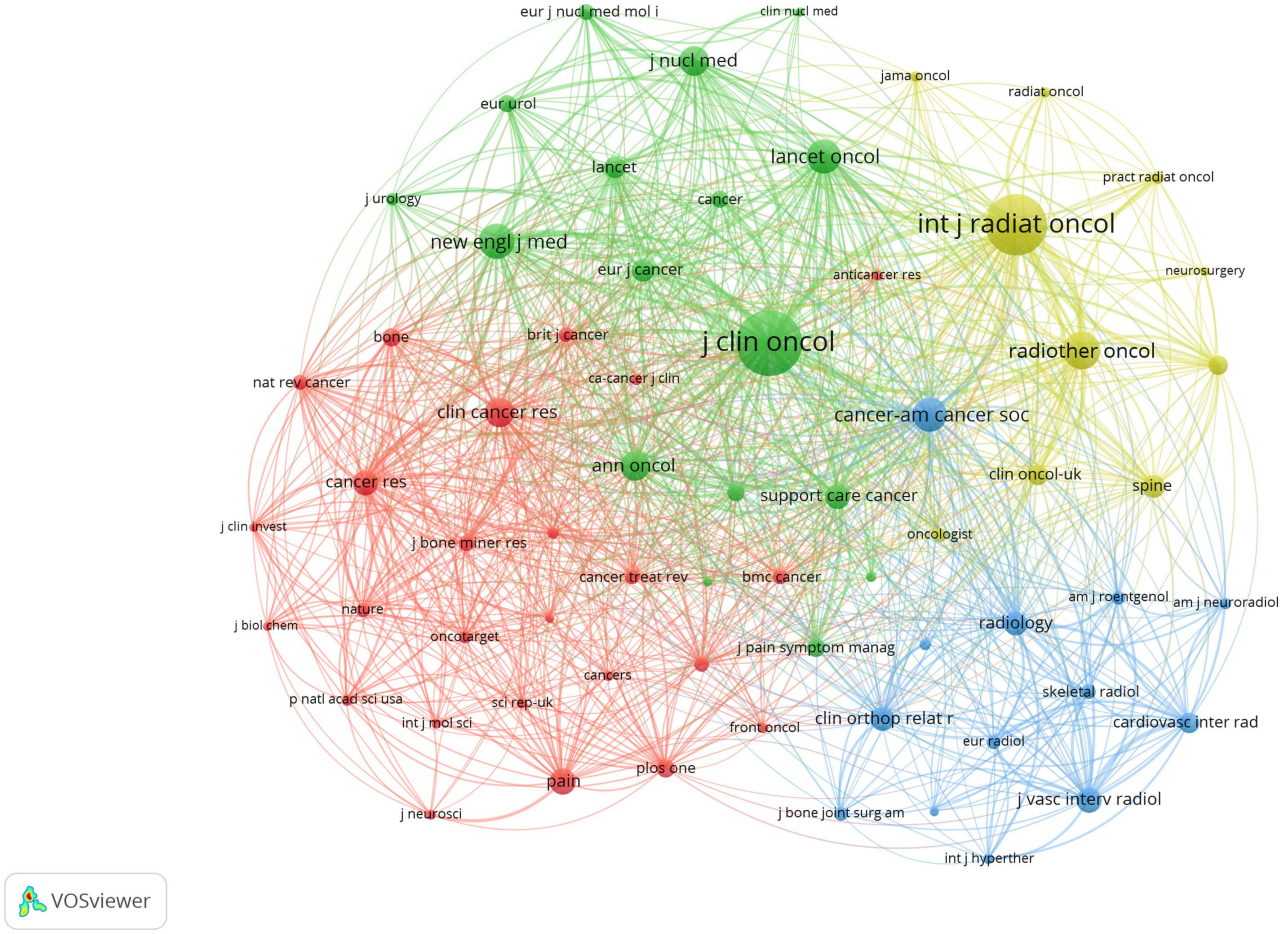
Journal co-citation network.

The journal co-citation network (Figure 11) intuitively displayed academic connections between these core knowledge sources. Four main clusters formed within the network:

Green cluster: Centred around top journals in clinical oncology and palliative care fields, such as “Journal of Clinical Oncology,” “Lancet Oncology,” “Annals of Oncology,” “Cancer-American Cancer Society” and “Supportive Care in Cancer”.

Yellow cluster: Mainly composed of authoritative journals in the field of radiation oncology, such as “International Journal of Radiation Oncology Biology Physics,” “Radiotherapy and Oncology” and “Practical Radiation Oncology”.

Red cluster: Included core journals in basic research, review-type journals, and pain and bone research, such as “Cancer Research,” “Nature Reviews Cancer,” “Clinical Cancer Research,” “Pain” and “Bone”.

Blue cluster: Gathered imaging and interventional radiology journals such as “Radiology,” “American Journal of Roentgenology,” “Skeletal Radiology,” “Journal of Vascular and Interventional Radiology” and “Cardiovascular and Interventional Radiology”.

These clusters clearly indicated that research on CIBP was profoundly influenced by developments in clinical oncology, radiation oncology, pain science, bone research, and related imaging and interventional techniques, with its knowledge foundation established upon authoritative journals in these core disciplines.

### 3.6 Analysis of references

Through co-citation analysis of references cited by the 1,822 included documents, we can identify key literature with foundational or milestone significance and the knowledge base in this field.

#### 3.6.1 Highly cited references

Citation frequency is an important indicator for measuring the influence of literature. Table 8 lists the top 15 references by citation frequency.

**Table 8:** Top 15 cited references in the dataset.

Citations	Year	Cited references
79	2013	Parker C, 2013, New Engl J Med, V369, P213, DOI 10.1056/NEJMoa1213755
64	2018	Rich SE, 2018, Radiother Oncol, V126, P547, DOI 10.1016/j.radonc.2018.01.003
61	2017	Lutz S, 2017, Pract Radiat Oncol, V7, P4, DOI 10.1016/j.prro.2016.08.001
54	2017	Macedo F, 2017, Oncol Rev, V11, P43, DOI 10.4081/oncol.2017.321
47	2012	Chow E, 2012, Clin Oncol-UK, V24, P112, DOI 10.1016/j.clon.2011.11.004
46	2014	Chow E, 2014, Lancet Oncol, V15, P164, DOI 10.1016/S1470-2045(13)70556-4
45	2014	Sartor O, 2014, Lancet Oncol, V15, P738, DOI 10.1016/S1470-2045(14)70183-4
43	2021	Sahgal A, 2021, Lancet Oncol, V22, P1023, DOI 10.1016/S1470-2045(21)00196-0
40	2021	Sung H, 2021, CA-Cancer J Clin, V71, P209, DOI 10.3322/caac.21660
38	2011	Lutz S, 2011, Int J Radiat Oncol, V79, P965, DOI 10.1016/j.ijrobp.2010.11.026
38	2019	Nguyen QN, 2019, JAMA Oncol, V5, P872, DOI 10.1001/jamaoncol.2019.0192
32	2018	Fornetti J, 2018, J Bone Miner Res, V33, P2099, DOI 10.1002/jbmr.3618
32	2011	Fizazi K, 2011, Lancet, V377, P813, DOI 10.1016/S0140-6736(10)62344-6
32	2020	Coleman RE, 2020, Nat Rev Dis Primers, V6, P0, DOI 10.1038/s41572-020-00216-3
32	2019	Smith M, 2019, Lancet Oncol, V20, P408, DOI 10.1016/S1470-2045(18)30860-X

Among these, the phase III clinical trial (ALSYMPCA study) on Radium-223 for treating castration-resistant prostate cancer with bone metastases, published by Parker C. et al. in 2013 in the New England Journal of Medicine, received the highest number of citations at 79. This was followed by Rich S.E. et al.’s (2018) systematic review of stereotactic body radiotherapy (SBRT) for spinal metastases (64 citations) and Lutz S. et al.’s (2017) updated ASTRO practice guideline for palliative radiotherapy (61 citations). Additionally, Macedo F. et al.’s (2017) review on bone metastasis diagnosis and treatment (54 citations), Chow E. et al.’s (2012) systematic review of radiotherapy for bone metastasis pain (47 citations), and Sung H. et al.’s (2021) GLOBOCAN global cancer statistics report (40 citations) were also amongst the highly cited literature. These highly cited references primarily encompassed key therapeutic approaches for bone metastases (such as Radium-223 and radiotherapy), clinical guidelines, epidemiological data, and important review studies, constituting the knowledge foundation widely referenced by researchers in this field.

#### 3.6.2 Reference co-citation network and cluster analysis

**Figure 12:**
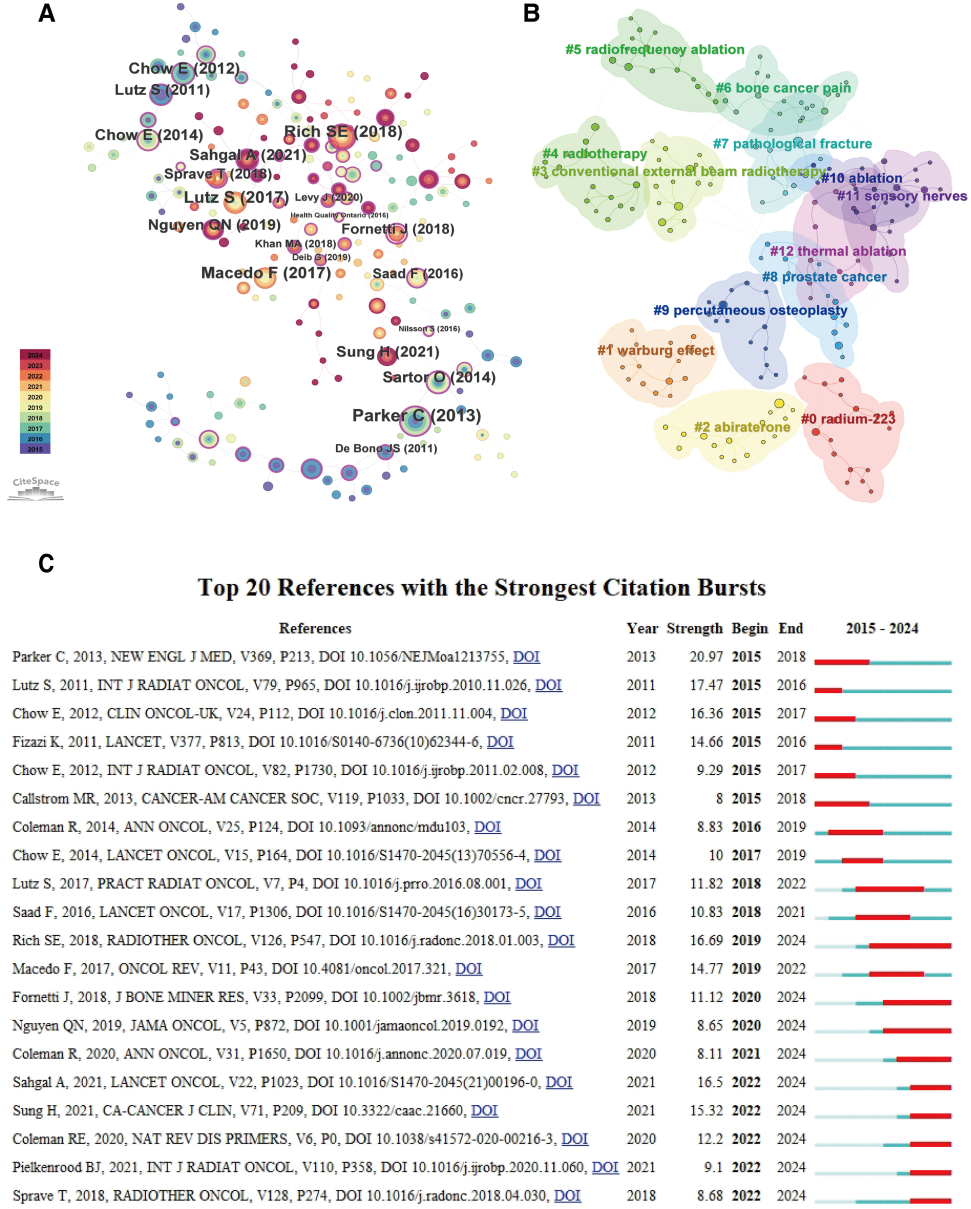
Reference co-citation analysis: network, clustering and burst detection. **(A)** Reference co-citation network diagram, where node size represents citation frequency, purple rings indicate high betweenness centrality, and colours represent the year of first citation. **(B)** Co-citation network diagram with overlay of cluster information, where labels represent cluster themes. **(C)** Reference citation burst timeline diagram, where red bars represent burst duration and intensity.

The reference co-citation network (Figure 12A) intuitively displayed knowledge associations between cited references.

In the network, each node represents a cited reference, with node size proportional to its citation frequency, and lines indicating the frequency of co-citation between references. Purple rings (Purple Ring) surrounding nodes indicate references with high betweenness centrality. Node colours represent the year of first citation (see legend), with warmer colours (such as red and orange) typically representing more recent literature. As shown in the figure, several interconnected reference groups formed around highly cited literature such as Parker C. (2013), Rich S.E. (2018), and Lutz S. (2017).

To further reveal the intrinsic themes of knowledge structure in this field, we used CiteSpace’s clustering function to analyse the co-citation network, identifying 13 major knowledge clusters (Figure 12B). Cluster labels were extracted from keywords of citing documents, representing core themes of the respective knowledge clusters. The larger clusters with clear structures included:

#0 radium-223: Focused on the application and efficacy of the radioisotope Radium-223 in treating prostate cancer bone metastases.

#1 warburg effect: Addressed potential connections between tumour metabolism (particularly the Warburg effect) and bone metastasis or pain mechanisms.

#2 abiraterone: Involved the role of the novel endocrine therapeutic agent abiraterone in treating prostate cancer bone metastases.

#3 conventional external beam radiotherapy / #4 radiotherapy: Encompassed the application, dose fractionation schemes, and efficacy comparisons of conventional external beam radiotherapy in palliative treatment of bone metastases.

#5 radiofrequency ablation / #10 ablation / #12 thermal ablation: Concentrated on the application of local ablative techniques such as radiofrequency ablation and thermal ablation in treating bone metastatic lesions and alleviating pain.

#6 bone cancer pain: Directly addressed the pathogenesis, assessment, and management of cancer-induced bone pain.

#7 pathological fracture: Explored the risk, prevention, and management of pathological fractures caused by bone metastases.

#8 prostate cancer: Focused on characteristics, treatment strategies, and prognosis of prostate cancer bone metastases.

#9 percutaneous osteoplasty: Concerned the application of percutaneous osteoplasty (vertebroplasty/kyphoplasty) in treating spinal metastases and relieving pain.

#11 sensory nerves: Explored the role and related mechanisms of sensory nerves in the development and maintenance of cancer-induced bone pain.

These clusters clearly delineated the major knowledge domains in research on CIBP, encompassing multiple dimensions from basic mechanisms (metabolism, neural), specific cancer types (prostate cancer), to diverse treatment approaches (radioisotopes, endocrine therapy, radiotherapy, ablation, surgery), as well as complications management (pathological fractures).

#### 3.6.3 Reference citation burst analysis

Reference citation burst analysis can identify literature with rapidly rising influence during specific time periods, revealing the dynamic evolution of research focuses. Figure 12C and Table 9 display the top 20 references by burst strength.

**Table 9:** Top 20 references by burst strength (2015–2024).

Begin	End	Strength	Year	Entity
2015	2018	20.9663	2015	Parker C, 2013, New Engl J Med, V369, P213, DOI 10.1056/NEJMoa1213755
2015	2016	17.4694	2015	Lutz S, 2011, Int J Radiat Oncol, V79, P965, DOI 10.1016/j.ijrobp.2010.11.026
2015	2017	16.364	2015	Chow E, 2012, Clin Oncol-UK, V24, P112, DOI 10.1016/j.clon.2011.11.004
2015	2016	14.6569	2015	Fizazi K, 2011, Lancet, V377, P813, DOI 10.1016/S0140-6736(10)62344-6
2015	2017	9.288	2015	Chow E, 2012, Int J Radiat Oncol, V82, P1730, DOI 10.1016/j.ijrobp.2011.02.008
2015	2018	7.9968	2015	Callstrom MR, 2013, CANCER-AM CANCER SOC, V119, P1033, DOI 10.1002/cncr.27793
2016	2019	8.8281	2015	Coleman R, 2014, Ann Oncol, V25, P124, DOI 10.1093/annonc/mdu103
2017	2019	10.0015	2015	Chow E, 2014, Lancet Oncol, V15, P164, DOI 10.1016/S1470-2045(13)70556-4
2018	2022	11.8206	2017	Lutz S, 2017, Pract Radiat Oncol, V7, P4, DOI 10.1016/j.prro.2016.08.001
2018	2021	10.8307	2016	Saad F, 2016, Lancet Oncol, V17, P1306, DOI 10.1016/S1470-2045(16)30173-5
2019	2024	16.6916	2018	Rich SE, 2018, Radiother Oncol, V126, P547, DOI 10.1016/j.radonc.2018.01.003
2019	2022	14.7728	2017	Macedo F, 2017, Oncol Rev, V11, P43, DOI 10.4081/oncol.2017.321
2020	2024	11.1245	2018	Fornetti J, 2018, J Bone Miner Res, V33, P2099, DOI 10.1002/jbmr.3618
2020	2024	8.649	2019	Nguyen QN, 2019, JAMA Oncol, V5, P872, DOI 10.1001/jamaoncol.2019.0192
2021	2024	8.1085	2020	Coleman R, 2020, Ann Oncol, V31, P1650, DOI 10.1016/j.annonc.2020.07.019
2022	2024	16.4974	2021	Sahgal A, 2021, Lancet Oncol, V22, P1023, DOI 10.1016/S1470-2045(21)00196-0
2022	2024	15.3184	2021	Sung H, 2021, CA-Cancer J Clin, V71, P209, DOI 10.3322/caac.21660
2022	2024	12.1953	2020	Coleman RE, 2020, Nat Rev Dis Primers, V6, P0, DOI 10.1038/s41572-020-00216-3
2022	2024	9.1024	2021	Pielkenrood BJ, 2021, Int J Radiat Oncol, V110, P358, DOI 10.1016/j.ijrobp.2020.11.060
2022	2024	8.6836	2018	Sprave T, 2018, Radiother Oncol, V128, P274, DOI 10.1016/j.radonc.2018.04.030

Results showed that Parker C. (2013)'s research on Radium-223 had the highest burst strength (20.97), with its burst period extending from 2015 to 2018, reflecting the widespread attention this study generated at that time. Early important literature on radiotherapy by Lutz S. (2011) and Chow E. (2012) also exhibited high-intensity bursts during 2015–2017. Notably, literature with rapidly rising influence in recent years (2022–2024 burst period) included: Rich S.E. (2018)'s research on SBRT (strength 16.69), Sahgal A. (2021)'s randomised controlled trial comparing SBRT with conventional radiotherapy (strength 16.50), Sung H. (2021)'s global cancer statistics report (strength 15.32), and Coleman R.E. (2020)'s review on bone metastases (strength 12.20). These recently bursting references indicated that the application of stereotactic body radiotherapy (SBRT), the latest data on global cancer burden, and in-depth understanding of bone metastasis mechanisms and management were current frontier directions of interest to researchers in this field.

#### 3.6.4 Reference betweenness centrality analysis

Betweenness centrality measures a reference’s bridging role in connecting different research topics or knowledge clusters. Table 10 lists the top 10 references by betweenness centrality.

**Table 10:** Top 10 references by betweenness centrality.

Centrality	Year	Cited references
0.83	2018	Fornetti J, 2018, J Bone Miner Res, V33, P2099, DOI 10.1002/jbmr.3618
0.72	2014	Sartor O, 2014, Lancet Oncol, V15, P738, DOI 10.1016/S1470-2045(14)70183-4
0.66	2016	Saad F, 2016, Lancet Oncol, V17, P1306, DOI 10.1016/S1470-2045(16)30173-5
0.65	2016	Nilsson S, 2016, Ann Oncol, V27, P868, DOI 10.1093/annonc/mdw065
0.58	2018	Khan MA, 2018, Am J Neuroradiol, V39, P1376, DOI 10.3174/ajnr. A5680
0.57	2020	Levy J, 2020, J Vasc Interv Radiol, V31, P1745, DOI 10.1016/j.jvir.2020.07.014
0.55	2016	Health Quality Ontario, 2016, Ont Health Technol ASSESS SER, V16, P1
0.54	2013	Parker C, 2013, New Engl J Med, V369, P213, DOI 10.1056/NEJMoa1213755
0.51	2019	Deib G, 2019, Am J Roentgenol, V212, P1377, DOI 10.2214/AJR.18.20386
0.48	2011	De Bono JS, 2011, New Engl J Med, V364, P1995, DOI 10.1056/NEJMoa1014618

Among these, Fornetti J. (2018)'s research on the role of extracellular vesicles in the bone metastasis microenvironment had the highest centrality (0.83), suggesting it might connect different research directions such as bone metastasis mechanisms, microenvironment, and potential therapeutic targets. Sartor O. (2014)'s literature on subsequent analysis of Radium-223 or prostate cancer treatment (centrality 0.72) and Saad F. (2016)'s research comparing denosumab with zoledronic acid for preventing skeletal-related events (centrality 0.66) also had high centrality, playing crucial bridging roles in connecting research on different treatment strategies (such as radioisotope therapy and bone-targeted drugs). These high-centrality references are critical for understanding the cross-disciplinary integration of knowledge in this field.

### 3.7 Analysis of keywords

To deeply reveal research hotspots, thematic structures and frontier dynamics in the field of CIBP, this study conducted co-occurrence, clustering and burst analyses of keywords from the included literature.

#### 3.7.1 High-frequency keywords and co-occurrence network

After statistical processing and consolidation, the top 15 keywords by frequency are shown in Table 11.

**Table 11:** Top 15 keywords by frequency.

Count	Keywords
637	Bone metastases
313	Bone metastasis
244	Management
241	Breast cancer
213	Quality of life
207	Survival
203	Cancer
202	Prostate cancer
186	Zoledronic acid
185	Disease
178	Radiation therapy
159	Double blind
146	Pain
131	Radiotherapy
130	Therapy
128	Palliative radiotherapy
108	Radiofrequency ablation

Beyond the core concepts used in the search—“bone metastases” (637 occurrences) and “pain” (146 occurrences)—keywords such as “management” (244 occurrences), “breast cancer” (241 occurrences), “quality of life” (213 occurrences), “survival” (207 occurrences), “cancer” (203 occurrences), “prostate cancer” (202 occurrences), “zoledronic acid” (186 occurrences), “disease” (185 occurrences) and “radiation therapy” (178 occurrences) constituted the core high-frequency keywords in this field, reflecting that research primarily revolved around the management of bone metastases, specific primary cancers (breast cancer, prostate cancer), patient outcomes (quality of life, survival) and key therapeutic approaches (zoledronic acid, radiotherapy).

**Figure 13:**
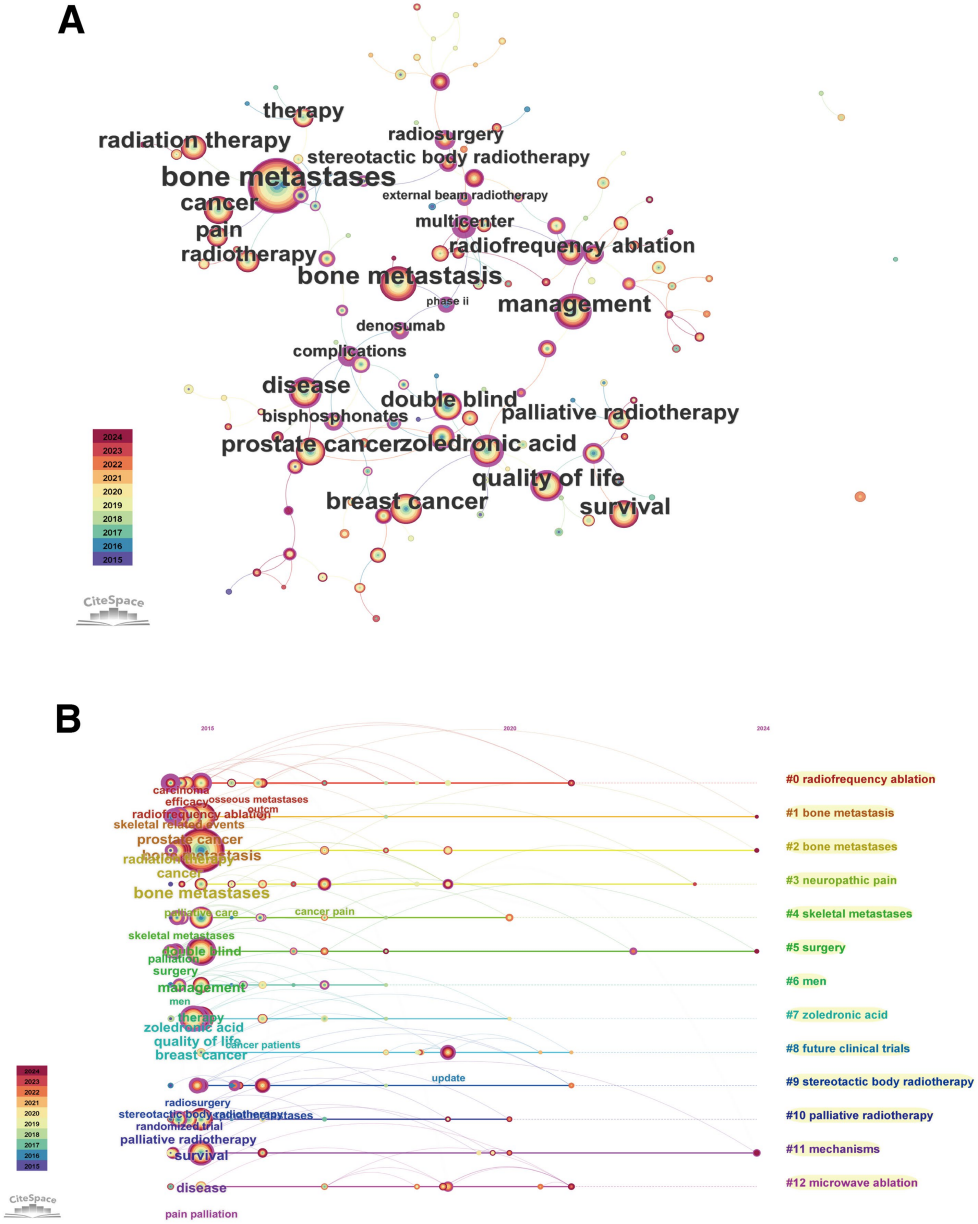
Keyword co-occurrence network and temporal evolution. **(A)** Keyword co-occurrence network diagram, where node size represents frequency and colour represents year of first appearance. **(B)** Keyword cluster timeline view, showing the evolution of major research themes (clusters) over time.

The keyword co-occurrence network was constructed using CiteSpace (Figure 13A). This network intuitively displayed the association strength and structural relationships between high-frequency keywords. Node size represented keyword occurrence frequency, node colour represented the year of first appearance (colour spectrum from purple to red indicating time from 2015 to 2024), and lines represented keywords co-occurring in the same document. As shown in the figure, high-frequency terms such as “bone metastases,” “pain,” “management,” “breast cancer” and “prostate cancer” were positioned at central or key locations in the network and formed close connections with other keywords.

Betweenness centrality analysis (a measure of a node’s importance as a bridge connecting different research themes) (Table 12) identified key nodes connecting different research themes.

**Table 12:** Top 20 keywords by betweenness centrality.

Centrality	Keywords
0.85	Complications
0.8	Multicenter
0.69	Phase ii
0.67	Denosumab
0.57	Zoledronic acid
0.51	Radiosurgery
0.49	External beam radiotherapy
0.43	Radiofrequency ablation
0.43	Bisphosphonates
0.39	Stereotactic body radiotherapy
0.35	Skeletal metastases
0.33	Tumors
0.32	Quality of life
0.31	Disease
0.31	Skeletal related events
0.31	Surgery
0.31	Solid tumors

Keywords such as “complications” (centrality 0.85) “multicenter” (0.80) “phase ii” (0.69) “denosumab” (0.67) and “zoledronic acid” (0.57) had the highest centrality indicating that research on clinical trials complications management and key drugs (denosumab zoledronic acid) played important bridging roles in connecting different research directions (such as different cancer types treatment modalities and outcome measures).

#### 3.7.2 Keyword cluster and temporal evolution

Based on the keyword co-occurrence network, CiteSpace automatically extracted and formed multiple research theme clusters. The keyword timeline view (Figure 13B) clearly displayed the major clusters and their evolutionary trajectories over time. In the figure, each horizontal band represented a cluster, nodes represented keywords, their positions indicated time of first appearance, and lines represented co-occurrence relationships. The major clusters and their core themes included:

#0 radiofrequency ablation: Focused on the application of radiofrequency ablation techniques in treating bone metastases.

#1 bone metastasis and #2 bone metastases: These two clusters revolved around the core concept of bone metastasis itself, spanning the entire research period and forming the foundation of research.

#3 neuropathic pain: Addressed mechanisms and characteristics of neuropathic pain induced by bone metastases, a theme with increasing activity in recent years.

#4 skeletal metastases: Similar to #1 and #2, emphasising the skeleton as the site of metastasis.

#5 surgery and #6 men: Possibly associated with surgical interventions for bone metastases and bone metastasis issues in male-specific cancers (such as prostate cancer), respectively.

#7 zoledronic acid: Represented research on anti-bone resorption drug therapy centred on zoledronic acid, which was particularly active in the early research period.

#8 future clinical trials: Focused on the design and direction of clinical trials in this field.

#9 stereotactic body radiotherapy and #10 palliative radiotherapy: Represented different radiotherapy techniques in bone metastasis pain management, with palliative radiotherapy research spanning throughout, whilst SBRT emerged as a newer hotspot.

#11 mechanisms: Addressed basic mechanism research on bone metastasis and related pain, with increasing activity in recent years.

#12 microwave ablation: Similar to #0, represented another minimally invasive ablation technique, a recent research hotspot.

The timeline view showed that attention to bone metastasis itself (#1, #2, #4) and palliative treatment (#10) was continuous. Research on early drugs represented by zoledronic acid (#7) still existed, but in recent years, the research focus had clearly shifted towards minimally invasive/interventional treatment techniques (#0, #9, #12), specific pain mechanisms (#3, #11) and future clinical trials (#8).

#### 3.7.3 Keyword burst detection and frontier analysis

**Figure 14:**
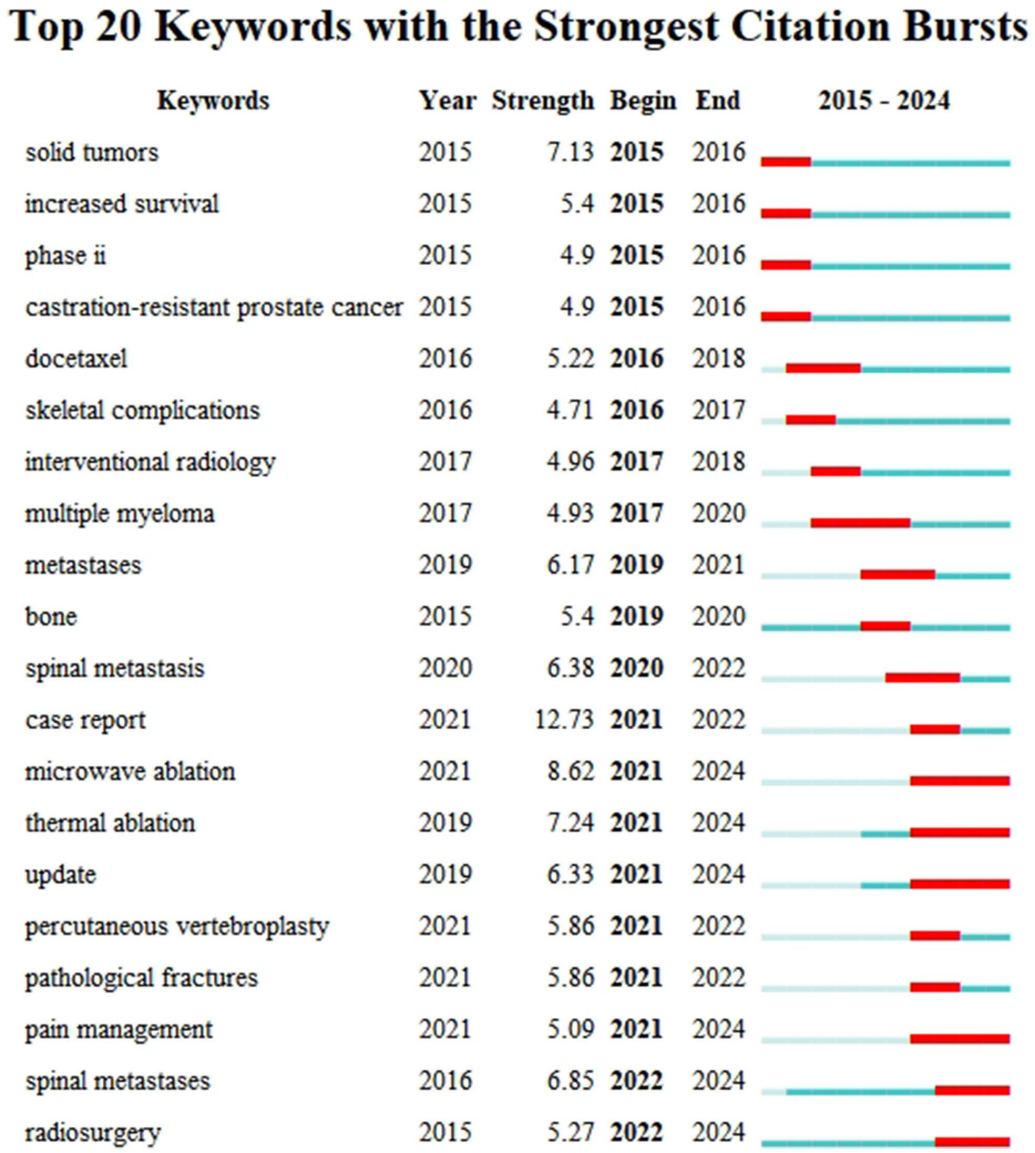
Keyword burst analysis (2015–2024).

Keyword burst analysis could identify frontier terms with rapidly increasing research interest during specific time periods (Figure 14; Table 13).

**Table 13:** Top 20 keywords by burst strength (2015–2024).

Begin	End	Strength	Year	Entity
2015	2016	7.1332	2015	Solid tumors
2015	2016	5.3965	2015	Increased survival
2015	2016	4.9048	2015	Phase ii
2015	2016	4.9048	2015	Castration-resistant prostate cancer
2016	2018	5.2168	2015	Docetaxel
2016	2017	4.7138	2015	Skeletal complications
2017	2018	4.9611	2015	Interventional radiology
2017	2020	4.9295	2015	Multiple myeloma
2019	2021	6.1673	2015	Metastases
2019	2020	5.4008	2015	Bone
2020	2022	6.384	2015	Spinal metastasis
2021	2022	12.7326	2015	Case report
2021	2024	8.6152	2015	Microwave ablation
2021	2024	7.2359	2015	Thermal ablation
2021	2024	6.3333	2015	Update
2021	2022	5.8583	2015	Percutaneous vertebroplasty
2021	2022	5.8583	2015	Pathological fractures
2021	2024	5.0932	2015	Pain management
2022	2024	6.8469	2015	Spinal metastases
2022	2024	5.2666	2015	Radiosurgery

Early burst terms (2015–2017) primarily included “solid tumors” (strength 7.13), “increased survival” (5.40), “phase ii” (4.90), “castration-resistant prostate cancer” (4.90), “docetaxel” (5.22) and “skeletal complications” (4.71), reflecting that research at that time focused on specific clinical trials (especially for prostate cancer), chemotherapeutic agents, and hard endpoints such as survival and complications.

Mid-term (2017–2020) burst terms included “interventional radiology” (4.96), “multiple myeloma” (4.93), “metastases” (6.17) and “bone” (5.40), indicating the expansion of research scope to other cancer types and interventional treatment fields.

In recent years (2021 to present), emerging burst keywords with higher intensity and longer duration have appeared, representing current research frontiers. These frontiers primarily concentrated on:

Minimally invasive/interventional and emerging radiotherapy techniques: “microwave ablation” (strength 8.62, 2021–2024), “thermal ablation” (7.24, 2021–2024), “radiosurgery” (5.27, 2022–2024).

Specific sites and clinical issues: “spinal metastases”/"spinal metastasis” (strength 6.85/6.38, 2022–2024/2020–2022), “pathological fractures” (5.86, 2021–2022).

New perspectives on pain management: The emergence of “pain management” (5.09, 2021–2024) might suggest increased attention to comprehensive and standardised pain management strategies.

Knowledge updates and reporting formats: The emergence of “update” (6.33, 2021–2024) and “case report” (12.73, 2021–2022) indicated rapid knowledge updates in this field and increased demand for sharing diagnostic and therapeutic experiences of special cases, respectively.

These burst keywords clearly delineated the research frontier landscape in the field of CIBP, showing a gradual shift from traditional drugs and survival endpoints towards minimally invasive interventional treatments, management of specific sites (such as the spine), pain management itself, and rapid knowledge updates.

In summary, bibliometric analysis clearly depicted that the research focus in the field of bone metastasis cancer pain is evolving from traditional drugs and endpoint indicators towards frontier directions including minimally invasive interventional treatments, specific pain mechanisms (such as neuropathic pain) and more comprehensive pain management. This transformation raises a core scientific question: whether common molecular regulatory networks exist behind these macroscopic research trends. To address this question, we subsequently employed bioinformatics analysis methods to explore core genes and pathways concurrently associated with both “bone metastasis” and “cancer pain” processes, with the aim of providing preliminary explanations and molecular-level clues for the macroscopic trends we observed.

### 3.8 Analysis of genes and pathways related to bone metastasis and associated pain

To explore potential mechanisms and key regulatory targets of bone metastasis and its associated pain at the molecular biology level, we conducted gene screening, protein–protein interaction (PPI) network construction and KEGG pathway enrichment analysis.

#### 3.8.1 Screening of core target genes

By searching the GeneCards database and screening genes with relevance scores greater than 20, we obtained 385 bone metastasis-related genes and 1,165 cancer pain-related genes. Intersection analysis of these two gene sets revealed 222 genes simultaneously associated with both bone metastasis and cancer pain. These intersection genes were considered potential core target genes involved in regulating the pathological process of CIBP, and were used for subsequent PPI network construction and pathway enrichment analysis.

#### 3.8.2 Analysis of protein–protein interaction network

**Figure 15:**
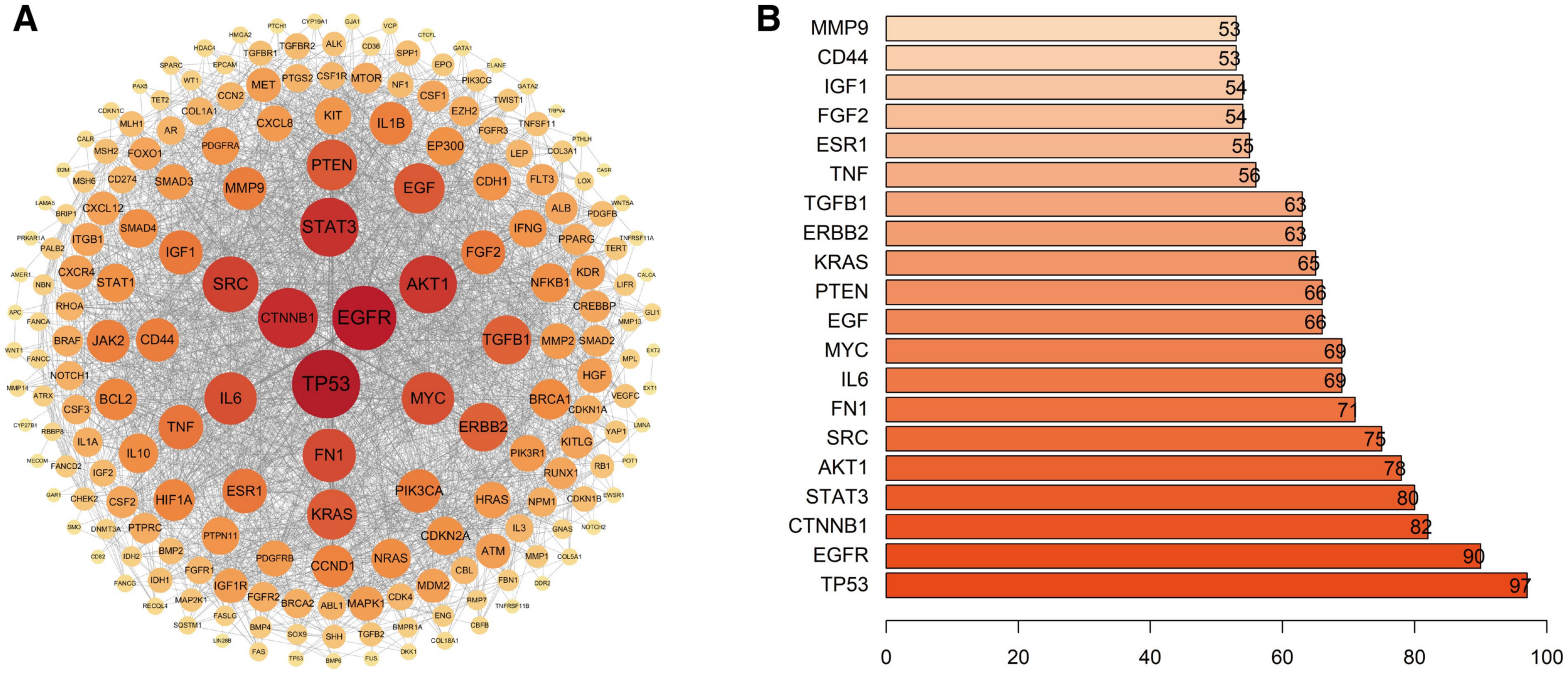
Protein–protein interaction (PPI) network of genes related to bone metastasis and cancer-induced pain. **(A)** PPI network constructed based on the STRING database (high confidence ≥0.7). **(B)** Top 20 core hub genes ranked by Degree centrality.

The 222 intersection genes were imported into the STRING database, with high confidence settings (minimum required interaction score = 0.700) and disconnected nodes hidden, to construct a protein–protein interaction (PPI) network (Figure 15A).

Cytoscape software was used to visualise the network and analyse node Degree centrality (Degree Centrality) to identify key hub proteins in the network. The top 20 core nodes by Degree centrality are shown in Figure 15B. Among these, TP53 (Degree = 97), EGFR (Degree = 90), CTNNB1 (Degree = 82), STAT3 (Degree = 80), AKT1 (Degree = 78), SRC (Degree = 75), FN1 (Degree = 71), IL6 (Degree = 69), MYC (Degree = 69) and EGF (Degree = 66) were the 10 genes with the highest Degree centrality. These core nodes had extremely high connectivity in the PPI network, suggesting they might play crucial roles in the complex regulatory network of bone metastasis and its associated pain.

#### 3.8.3 KEGG pathway enrichment analysis

To further explore the biological functions and signalling pathways involving these 222 core target genes, we conducted KEGG pathway enrichment analysis using the clusterProfiler package on the R platform. The results identified more than 100 significantly enriched pathways (FDR < 0.05).

Overall, these core genes were significantly enriched in several key pathway categories:

Cancer-related pathways: Such as Proteoglycans in cancer, MicroRNAs in cancer, Pathways in cancer, and various specific cancer pathways (such as Prostate cancer, Gastric cancer, Bladder cancer, Breast cancer, Hepatocellular carcinoma, Colorectal cancer, etc.).

Cell signal transduction pathways: Such as PI3K-Akt signalling pathway, MAPK signalling pathway, Ras signalling pathway, Rap1 signalling pathway, JAK–STAT signalling pathway, ErbB signalling pathway, HIF-1 signalling pathway, etc.

Cellular process pathways: Such as Cellular senescence, Focal adhesion, Regulation of actin cytoskeleton, etc.

Immune and inflammation-related pathways: Such as AGE-RAGE signalling pathway in diabetic complications, Cytokine-cytokine receptor interaction, TNF signalling pathway, IL-17 signalling pathway, etc.

Endocrine and metabolic pathways: Such as FoxO signalling pathway, Thyroid hormone signalling pathway, Relaxin signalling pathway, etc.

These results indicated that the development and progression of CIBP involved complex molecular network regulation, closely associated with multiple biological processes including cancer progression, key signal transduction, cellular senescence, inflammatory immune responses, and endocrine metabolism.

**Figure 16:**
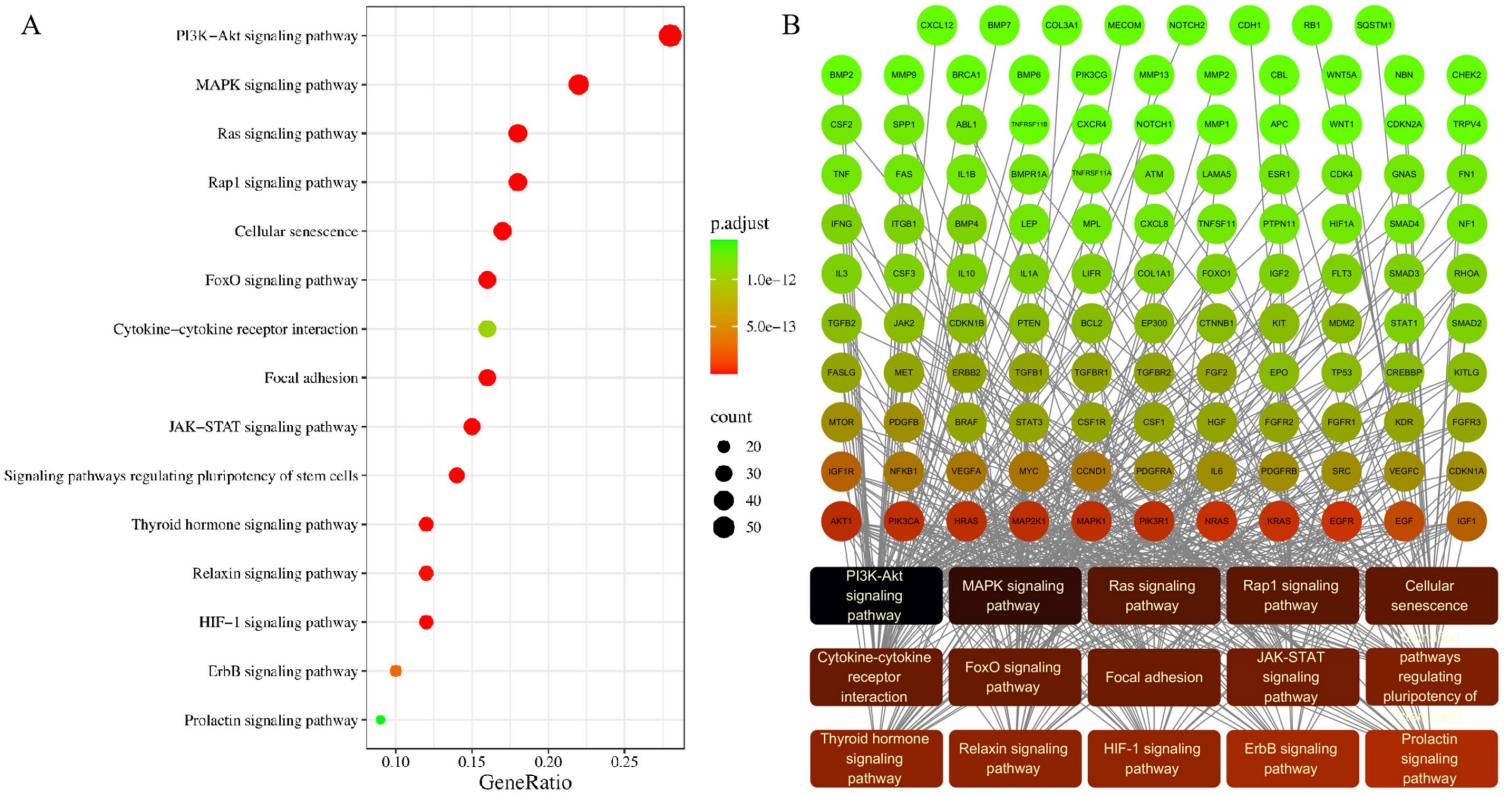
Core gene-pathway regulatory network derived from KEGG enrichment analysis. **(A)** Bubble plot of the top 15 enriched signalling pathways, where bubble size reflects the gene count and the colour represents the adjusted *p*-value. **(B)** Network visualisation of the regulatory connections between core genes and these key pathways.

Finally, we constructed a regulatory network between core genes and the top 15 enriched signalling pathways (after excluding human disease pathways) (Figure 16).

This network intuitively demonstrated how core genes such as TP53, EGFR, AKT1, STAT3, MYC and IL6 participated in regulating multiple key signalling pathways (such as PI3K-Akt, MAPK, JAK–STAT, etc.), highlighting the central position and potential therapeutic target value of these genes in the molecular mechanism network of bone metastasis and bone metastasis and its associated pain.

## 4 Discussion

Tumour bone metastasis and its accompanying intractable pain (CIBP) constitute formidable clinical challenges for advanced cancer patients, severely compromising quality of life whilst posing significant threats to treatment response and overall survival (12). Despite sustained attention from clinical and basic research communities and evolving therapeutic approaches, the complex pathophysiological mechanisms of CIBP remain incompletely elucidated, resulting in frequently inadequate clinical pain control and therapeutic limitations (12, 13). Confronted with the exponential growth of research literature over the past decade (2015–2024), traditional literature review methods struggle to comprehensively and objectively grasp the overall knowledge structure, evolutionary trajectories and potential research frontiers within this complex field, facing issues of cognitive fragmentation and temporal lag. To systematically navigate this rapidly developing domain and identify key research hotspots and knowledge gaps, our study innovatively combined bibliometric and bioinformatic approaches, aiming to create a data-driven, panoramic scientific map providing macro-level insights and valuable references for researchers and clinicians in this field.

Our analysis first revealed the robust vitality and continuously growing academic influence of the CIBP field over the past decade. Whilst annual publication volumes fluctuated, they generally maintained high levels, whilst annual total citation frequencies showed a steep upward trend (Figure 2), clearly indicating increasing global academic attention to CIBP issues, with growing research investment and heightened focus on relevant findings. Behind this growth trend lies the dual driving forces of urgent clinical demand for more effective pain management strategies and advances in basic research (such as deeper understanding of pain mechanisms) and clinical technologies (such as novel imaging, precision radiotherapy and targeted drugs). This growth was not uniformly distributed but closely correlated with the evolution of research frontiers revealed through our keyword and literature co-citation analyses (Figures 12–14). Early research (2015–2017) was predominantly focused on traditional drugs such as “zoledronic acid” and survival endpoints, whereas in recent years, with deepening understanding of CIBP mechanisms, research interest has shifted significantly towards more precise and less invasive therapeutic approaches, such as “stereotactic body radiotherapy (SBRT)” and “microwave ablation,” as well as greater attention to patient experience through “quality of life” and “pain management.” This indicates that academic output growth in this field has been driven by the dual forces of clinical demand and technological innovation, evolving from the fundamental question of “how to extend survival” to the more sophisticated clinical challenge of “how to achieve high-quality pain control whilst extending survival”.

Regarding the global research landscape, our study identified a typical core-periphery structure, with research strength highly concentrated in a few countries and top institutions (Tables 2, 3; Figures 4, 5). The United States, with its rich scientific foundation and extensive collaboration network, maintained absolute leadership in this field, outpacing others in publication quantity, academic influence, and breadth and intensity of international collaboration. China, as a significant contributor, has risen to second place in publication volume with rapid recent growth (Figure 3B), demonstrating enormous research potential. Particularly noteworthy is that China has not only achieved catch-up in publication numbers, even surpassing the United States in 2022 (Figure 3B), but its research content also reflects rapid advancement in emerging fields. For instance, in our keyword burst analysis, terms representing the frontier of minimally invasive interventional treatments, such as ‘microwave ablation’ and ‘thermal ablation’, exhibited high-intensity bursts after 2021 (Figure 14), whilst institutional analysis reveals (Figure 5A; Table 3) that leading Chinese institutions such as Shanghai Jiao Tong University have contributed significant output in this field. This indicates that Chinese scholars are not merely catching up but are demonstrating robust innovative vitality and clinical translation capabilities in emerging, technology-driven therapeutic domains. Whilst its international collaboration network, as indicated by a Total Link Strength of 85, is still developing compared to Western countries such as the USA, UK and Canada (Table 2; Figure 4). The outstanding contributions of the University of Toronto in Canada and its core scholar Edward Chow in this field were particularly noteworthy (Tables 3, 4; Figures 5A, 7A), possibly linked to their long-standing leadership in palliative radiotherapy and clinical practice guideline development. Institutional and author-level collaboration network analyses (Figures 6, 7B) further revealed that whilst transnational collaboration existed, closer collaboration typically occurred within the same country, region, or amongst teams with similar research directions. This pattern suggests the future need to further break down barriers and promote broader, more balanced international exchange and substantive collaboration, particularly strengthening integration between teams from different disciplinary backgrounds (such as basic and clinical, different treatment approaches) to jointly address CIBP as a global challenge.

Journal analysis results (Tables 6, 7; Figures 9–11) fully substantiated the multidisciplinary cross-cutting nature of CIBP research. Relevant research outcomes were widely published in professional journals across multiple fields, including pain science (such as “Pain”), clinical oncology (such as “J Clin Oncol”), radiation oncology (such as “Int J Radiat Oncol”), bone research (such as “J Bone Oncol”), palliative medicine (such as “Supportive Care in Cancer”) and interventional radiology. Notably, journals in clinical oncology and radiation oncology constituted the core citation sources for the knowledge system in this field, indicating that the demands of clinical practice and optimisation of treatment strategies were the central driving forces behind research development in this domain.

The most significant contribution of this study lies in our ability to establish logical connections between macroscopic research trends and microscopic molecular mechanisms through the integration of bibliometrics and bioinformatics. Bibliometric analysis clearly tracked the dynamic evolution of research focus (Figures 12–14; Tables 10–13), with the core transition being from early emphasis on traditional drugs and survival endpoints to a significant shift towards precision treatments represented by ‘stereotactic body radiotherapy (SBRT)’ and ‘microwave ablation’, whilst deepening exploration of ‘neuropathic pain’ as a fundamental pathological mechanism.

This shift in research emphasis is not an isolated phenomenon but demonstrates profound correspondence with the core molecular networks revealed through our bioinformatics analysis. Our analysis identified signalling pathways including TP53, EGFR, PI3K-Akt, MAPK, IL-6/STAT3 and TNF-*α* as key nodes connecting bone metastasis and pain (Figure 16). The emergence of precision radiotherapy and minimally invasive interventional techniques precisely corresponds to pathways such as PI3K-Akt, MAPK and TNF-α, which regulate cellular survival, radiosensitivity and inflammatory responses. Simultaneously, in-depth exploration of neuropathic pain mechanisms directly corresponds to the central roles of EGFR, IL-6/STAT3 and MAPK pathways in neuronal sensitisation and neuroglial cell activation. The functional status of TP53 serves as an upstream master switch determining tumour invasive potential, providing the foundation for all these downstream pathological processes.

This correspondence reveals the intrinsic logic underlying research evolution in the CIBP field: advances in clinical practice are synchronous with, and mutually drive, our deepening understanding of fundamental molecular mechanisms. For instance, the rapid advancement of Chinese scholars in emerging minimally invasive interventional technologies (Figure 5A) exemplifies this close integration of basic science and clinical practice, demonstrating enormous innovative potential. Therefore, the integrated analytical framework from macroscopic to microscopic constructed in this study provides clear entry points for our subsequent in-depth exploration of each pathway’s specific role in CIBP (as detailed in the following text), ultimately directing all clues towards mechanism-based, multi-target combination therapeutic strategies as the future direction.

The bioinformatic analysis introduced in this study provided a unique complementary perspective for understanding CIBP comorbidity mechanisms at the molecular level. The 222 core genes shared between bone metastasis and cancer pain that we identified (Figure 14) and their complex PPI network (Figure 15) revealed a highly correlated molecular interaction system. The bioinformatics analysis in this study identified 222 shared genes connecting bone metastasis and pain. The protein–protein interaction network formed by these genes (Figure 15) revealed that core genes such as TP53 and EGFR, along with their associated signalling pathways including IL-6/STAT3, TNF-*α*, PI3K-Akt and MAPK, are the most highly connected and functionally central molecular nodes. These pathways do not operate independently but form a highly integrated pathological network that collectively drives cancer-induced bone pain (CIBP), a complex syndrome exhibiting both nociceptive and neuropathic characteristics.

The functional status of TP53 serves as an upstream switch determining tumour invasive potential. Wild-type p53 actively suppresses tumour metastasis through regulation of cellular adhesion, motility and anoikis processes (14). However, in most cancers, missense mutations in TP53 not only result in loss of its tumour suppressor function (Loss-of-Function, LOF) but also confer new oncogenic gain-of-function (GOF) properties (15). GOF-mutant p53 actively promotes epithelial-mesenchymal transition (EMT) and receptor tyrosine kinase (RTK) signalling, endowing tumour cells with enhanced invasive and metastatic capabilities (15). Therefore, whilst the mutational status of TP53 does not directly regulate neuronal excitability, it serves as a fundamental, upstream molecular event determining whether tumours can successfully invade bone, destroy tissues and trigger all subsequent pain-inducing events, creating the pathological prerequisite for CIBP occurrence.

EGFR signalling represents the central hub connecting tumour growth and pain transmission. Aberrant activation of the EGFR pathway is a driving force behind the “vicious cycle” of bone metastasis, stimulating tumour cells to produce osteolytic factors and regulating the RANKL/OPG ratio to promote osteoclastogenesis (16, 17). More importantly, EGFR directly participates in pain signal generation. EGFR and its ligands are expressed on sensory neurons in the dorsal root ganglia (DRG), and their activation can directly drive pain hypersensitivity whilst exhibiting crosstalk with NMDAR, a key molecule in central sensitisation (18, 19). This dual functionality—driving tumour pathology whilst directly regulating pain symptoms—makes EGFR a unique therapeutic target, with its inhibitors potentially possessing both disease-modifying and direct analgesic effects.

The inflammatory pathways IL-6/STAT3 and TNF-*α* represent common driving forces for bone destruction and neuroinflammation. In bone metastatic sites, IL-6 forms autocrine/paracrine loops between tumour cells and bone marrow stromal cells through the JAK/STAT3 axis, promoting tumour growth, angiogenesis and bone destruction (20, 21). Simultaneously, IL-6, as a core algogenic cytokine, can directly induce neuroinflammation and sensitisation in both peripheral and central nervous systems (22, 23). TNF-α, as a potent inducer of bone lysis, directly promotes osteoclastogenesis whilst inhibiting osteoblast function (24, 25). It is also a recognised algogenic mediator capable of directly sensitising nociceptors and driving peripheral and central neuroinflammation (26). More specifically, at the central level, TNF-α released by activated glial cells can enhance excitatory synaptic transmission and inhibit inhibitory signals through its TNFR1 receptor, thereby actively remodelling pain circuits (27, 28). These two pathways function as pathological amplifiers, transforming localised bone metastatic sites into sustained, self-amplifying neuroinflammatory states, which are key to the chronicity and intractability of CIBP.

The enrichment analysis in this study equally highlighted the importance of the IL-17 pathway. Traditionally, IL-17 was considered to be primarily produced by peripheral Th17 cells, promoting bone destruction through indirect mechanisms (such as inducing IL-6) (29). However, the bioinformatics analysis results from this study align with recent breakthrough research, collectively revealing its more direct and central role in CIBP. Under the stimulation of bone metastasis, spinal astrocytes become the primary source of IL-17A production, directly acting on neurons expressing IL-17RA receptors and activating downstream CaMKIIα signalling, thereby driving central sensitisation and pain (30). This newly discovered “astrocyte-IL-17A-neuron” signalling axis reveals that intrinsic neuroinflammation within the central nervous system can serve as a direct driver of pain, providing a novel perspective for understanding the intractability of CIBP and suggesting that targeting central IL-17 signalling may represent a highly promising future therapeutic strategy.

The PI3K-Akt and MAPK pathways function as core processors for cellular survival and neural plasticity. These two pathways serve as common downstream convergence points for multiple upstream signals including EGFR, IL-6 and TNF-α. The PI3K-Akt pathway drives the “Warburg effect,” leading to massive lactate accumulation in the tumour microenvironment and creating an acidic milieu, which itself constitutes a powerful noxious stimulus capable of directly activating acid-sensitive ion channels (such as ASIC3 and TRPV1) on sensory nerve terminals (31). Meanwhile, the PI3K-Akt–mTOR pathway is also a key regulator of central sensitisation and synaptic plasticity (22, 32). The MAPK/ERK pathway plays a central “cellular relay” role in the chronification process of CIBP, with its activity sequentially transmitted in the spinal cord following a “neuron → microglia → astrocyte” pattern, clearly depicting the transformation of pain from acute nociceptive signals to chronic neuropathic states maintained by glial cells (33).

Taken together, these pathways constitute a highly robust, interconnected signalling network. Upstream receptors such as EGFR integrate growth and inflammatory signals, distributing them to core intracellular processors such as PI3K-Akt and MAPK, ultimately resulting in a series of pathophysiological consequences including bone destruction, acidosis, neuroinflammation and neural plasticity changes. The redundancy and feedback characteristics of this network explain why treatments targeting single nodes often yield limited efficacy. This provides clear guidance for future therapeutic strategies, indicating that combination therapies capable of simultaneously intervening at multiple critical nodes must be employed to thoroughly dismantle this pathological network and achieve effective control of CIBP.

Therefore, future experimental research should no longer view bone metastasis and pain in isolation. For instance, when evaluating a novel anti-tumour drug, in addition to traditional tumour volume indicators, routine assessment of pain behavior and neuroinflammatory markers should be incorporated (34). Conversely, when developing novel analgesic strategies, their effects on tumour cell proliferation and bone destruction within the bone metastatic microenvironment should also be evaluated. Core genes identified in this study, such as TP53 and EGFR, could serve as potential biomarkers for patient selection, efficacy prediction and disease progression monitoring in future clinical trials. Particularly, China possesses a vast patient population and an increasingly sophisticated clinical research system, conferring unique advantages and enormous developmental potential in advancing such translational research that integrates basic and clinical, oncological and pain-related approaches. Overall, promoting this integrative research paradigm will be the essential pathway for conquering CIBP as a clinical challenge globally.

These findings spanning from macroscopic trends to microscopic mechanisms hold important guiding significance for CIBP clinical practice. Firstly, at the diagnostic and assessment level, the emergence of “neuropathic pain” (Figure 13B, Cluster #3) as an independent research theme revealed in this study, corroborated by the enrichment of neuroinflammation-related pathways such as TNF and IL-17 (Figure 16), suggests that clinicians should routinely employ specialised tools such as DN4 for precise pain phenotyping when assessing CIBP, thereby providing evidence for the addition of drugs targeting neuropathic pain (35, 36). Secondly, regarding treatment strategies, the citation burst trends of SBRT and thermal ablation techniques (Figure 12C; Table 9) support the clinical shift towards localised precision therapy. The PI3K-Akt and other pathways identified in this study, which are associated with radiosensitivity, provide theoretical foundations for designing clinical trials combining SBRT with targeted drugs (37, 38). Notably, China’s contributions in this field are rapidly increasing (Figure 3B), particularly in emerging technologies such as microwave ablation (Figure 14), where Chinese scholars have published substantial high-quality clinical research, accumulating valuable evidence-based medical evidence for the global application of these technologies. Finally, the shared pathways identified in this study, such as EGFR and IL-6/STAT3, point towards developing dual-effect therapies capable of simultaneously controlling tumours and alleviating pain, which holds promise for fundamentally transforming the therapeutic landscape of CIBP.

Certainly, whilst interpreting these findings, we must acknowledge limitations of this study. Firstly, regarding data source limitations, this study included only literature from the Web of Science Core Collection, potentially omitting important research from other databases (such as PubMed, Scopus) or non-English publications, which may introduce certain bias in depicting the global research landscape. Secondly, there are inherent limitations of bibliometric indicators, as citation counts and publication volumes cannot fully reflect the intrinsic quality and innovativeness of research. Thirdly, the accuracy of keyword analysis is constrained by author annotations and database indexing, potentially failing to capture all relevant concepts, particularly emerging terminology. Finally, and critically important, is the predictive nature of bioinformatics analysis. The bioinformatics component of this study is essentially association mining and prediction based on existing knowledge repositories. For instance, we employed a GeneCards relevance score greater than 20 as our screening criterion—a commonly used threshold aimed at balancing sensitivity and specificity—yet this may still overlook certain low-scoring genes with potential significance. Whilst the core genes and pathways it identified provide valuable clues for subsequent research, the causal relationships and specific functions between them still require extensive, in-depth experimental research for confirmation and elucidation. Therefore, the bioinformatics findings of this study should be regarded as exploratory, intended to provide direction for future wet laboratory research.

Despite these limitations, the systematic analysis of this study still provides clear directional insights for future development in the field of CIBP. Future research should continue to deepen exploration of complex CIBP mechanisms, particularly focusing on molecular pathways of neuropathic pain, fine interactions between the immune microenvironment and nervous system, and functional validation of core genes (such as TP53, EGFR, IL6, STAT3) and pathways (such as PI3K-Akt, MAPK, TNF) predicted in this study. Regarding clinical research, more rigorously designed trials are needed to evaluate the efficacy, safety and long-term impact on quality of life of SBRT, ablation techniques, novel targeted drugs, immunotherapy and optimised combinations of these approaches in different CIBP subtype patients. Strengthening the translation between basic research and clinical practice, applying molecular mechanism discoveries to the development of new diagnostic markers or therapeutic targets is crucial. Simultaneously, the patient-centred concept should be continuously promoted, pain assessment systems improved (especially for neuropathic components), multidisciplinary collaboration models strengthened, and individualised comprehensive pain management realised. Finally, we call for establishing broader and deeper international collaboration platforms, promoting resource sharing and idea exchange amongst researchers from different countries and disciplinary backgrounds, to collaboratively tackle CIBP as a global health challenge.

## 5 Conclusion

This study, for the first time combining bibliometric and bioinformatic methods, systematically depicted the global research landscape of the CIBP field over the past decade (2015–2024). The analysis revealed the field’s continuously growing academic vitality and a critical shift in research focus from traditional palliative treatments towards neuropathic pain mechanisms, immune microenvironments, and precision treatment strategies represented by SBRT and minimally invasive ablation. Additionally, this study identified core contributing countries, institutions and scholar networks, whilst bioinformatic analysis pinpointed potential core genes (such as TP53, EGFR, IL6) and key signalling pathways (such as PI3K-Akt, MAPK, TNF) connecting bone metastasis and pain. These findings collectively constitute a data-driven knowledge map that not only clearly displays the evolutionary trajectory and frontier hotspots of this interdisciplinary field but also provides important macro-level guidance for future researchers to identify knowledge gaps, seek collaborations, and explore new molecular targets. In sum, this study offers valuable, evidence-based references for advancing both basic research and clinical translation in tumour bone metastatic pain.

## Data Availability

The literature data used in this analysis were obtained from publicly available databases: the Web of Science Core Collection (WoSCC). WoSCC is a subscription database accessible through institutional subscriptions. Intermediate datasets used to generate analytical results in this study (such as filtered literature lists, network analysis files, gene lists, etc.) and related code are available from the corresponding authors upon reasonable request. Gene and pathway information obtained from GeneCards, STRING and KEGG databases are all publicly available data.

## Footnotes

^1^ https://www.webofscience.com/wos/woscc/

^2^ https://www.genecards.org/

^3^ http://www.bioinformatics.com.cn/

^4^ https://cn.string-db.org/

^5^ https://cytoscape.org/

^6^ https://r-project.org

## References

[1] Clézardin P Coleman R Puppo M Ottewell P Bonnelye E Paycha F . Bone metastasis: mechanisms, therapies, and biomarkers. Physiol Rev. (2021) 101:797–855. doi: 10.1152/physrev.00012.2019 33356915

[2] Ryan C Stoltzfus KC Horn S Chen H Louie AV Lehrer EJ . Epidemiology of bone metastases. Bone. (2022) 158:115783. doi: 10.1016/j.bone.2020.115783 33276151

[3] Kang J La Manna F Bonollo F Sampson N Alberts I Mingels C . Tumor microenvironment mechanisms and bone metastatic disease progression of prostate cancer. Cancer Lett. (2022) 530:156–69. doi: 10.1016/j.canlet.2022.01.015 35051532

[4] Takei D Tagami K. Management of cancer pain due to bone metastasis. J Bone Miner Metab. (2023) 41:327–36. doi: 10.1007/s00774-022-01382-y 36418587

[5] Jing D Zhao Q Zhao Y Lu X Feng Y Zhao B . Management of pain in patients with bone metastases. Front Oncol. (2023) 13:1156618. doi: 10.3389/fonc.2023.1156618 37007073 PMC10063159

[6] Yang L Liu B Zheng S Xu L Yao M. Understanding the initiation, delivery and processing of bone cancer pain from the peripheral to the central nervous system. Neuropharmacology. (2023) 237:109641. doi: 10.1016/j.neuropharm.2023.109641 37392821

[7] Yang Y Yang W Zhang R Wang Y. Peripheral mechanism of cancer-induced bone pain. Neurosci Bull. (2024) 40:815–30. doi: 10.1007/s12264-023-01126-6 37798428 PMC11178734

[8] Sliepen SHJ. Bone cancer pain, mechanism and treatment. In: A Hiran, editor. Recent advances in bone Tumours and osteoarthritis. London, UK: Intech Open (2021)

[9] Wang X Li L Wang Y. Mechanisms of cancer-induced bone pain. J Pain Res. (2025) 18:315–26. doi: 10.2147/JPR.S498466 39867539 PMC11760761

[10] Oliveira O Silva F Juliani F Barbosa L Nunhes T Oliveira O . Bibliometric method for mapping the state-of-the-art and identifying research gaps and trends in literature: An essential instrument to support the development of scientific projects. In: K Suad Z Enver, editors. Scientometrics Recent Advances. London, UK: Intech Open (2019).

[11] Gencer G Gencer K. Large language models in healthcare: a bibliometric analysis and examination of research trends. J Multidiscip Healthc. (2025) 18:223–38. doi: 10.2147/JMDH.S502351 39844924 PMC11750729

[12] Zajączkowska R Kocot-Kępska M Leppert W Wordliczek J. Bone pain in cancer patients: mechanisms and current treatment. Int J Mol Sci. (2019) 20:6047. doi: 10.3390/ijms20236047 31801267 PMC6928918

[13] Middlemiss T Laird BJA Fallon MT. Mechanisms of cancer-induced bone pain. Clin Oncol. (2011) 23:387–92. doi: 10.1016/j.clon.2011.03.003 21683564

[14] Powell E Piwnica-Worms D Piwnica-Worms H. Contribution of p 53 to metastasis. Cancer Discov. (2014) 4:405–14. doi: 10.1158/2159-8290.CD-13-0136 24658082 PMC4063123

[15] Tang Q Su Z Gu W Rustgi AK. Mutant p 53 on the path to metastasis. Trends Cancer. (2020) 6:62–73. doi: 10.1016/j.trecan.2019.11.004 31952783 PMC7485681

[16] Mohammad KS Akhund SA. From tumor to bone: growth factor receptors as key players in cancer metastasis. Front Biosci (Landmark Ed). (2024) 29:184. doi: 10.31083/j.fbl2905184 38812320

[17] Foley J Nickerson N Nam S Nickerson NK Allen KT Gilmore JL . EGFR signaling in breast cancer: bad to the bone. Semin Cell Dev Biol. (2010) 21:951–60. doi: 10.1016/j.semcdb.2010.08.009 20813200 PMC2991402

[18] Santi MD Zhang M Liu N Viet CT Xie T Jensen DD . Repurposing EGFR inhibitors for oral cancer pain and opioid tolerance. Pharm (basel Switz). (2023) 16:1558. doi: 10.3390/ph16111558 PMC10674507 38004424

[19] Borges JP Mekhail K Fairn GD Antonescu CN Steinberg BE. Modulation of pathological pain by epidermal growth factor receptor. Front Pharmacol. (2021) 12:642820. doi: 10.3389/fphar.2021.642820 34054523 PMC8149758

[20] Ara T Declerck YA. Interleukin-6 in bone metastasis and cancer progression. Eur J Cancer. (2010) 46:1223–31. doi: 10.1016/j.ejca.2010.02.026 20335016 PMC2917917

[21] Huang B Lang X Li X. The role of IL-6/JAK2/STAT3 signaling pathway in cancers. Front Oncol. (2022) 12:1023177. doi: 10.3389/fonc.2022.1023177 36591515 PMC9800921

[22] Zhang J Wang L Wang H Su Z Pang X. Neuroinflammation and central PI3K/akt/mTOR signal pathway contribute to bone cancer pain. Mol Pain. (2019) 15:1744806919830240. doi: 10.1177/1744806919830240 30717619 PMC6390230

[23] Zhou Y Liu Z Liu Z Chen S Li M Shahveranov . Interleukin-6: an emerging regulator of pathological pain. J Neuroinflammation. (2016) 13:141. doi: 10.1186/s12974-016-0607-6 27267059 PMC4897919

[24] Aielli F Ponzetti M Rucci N. Bone metastasis pain, from the bench to the bedside. Int J Mol Sci. (2019) 20:280. doi: 10.3390/ijms20020280 30641973 PMC6359191

[25] Kitaura H Marahleh A Ohori F Noguchi T Nara Y Pramusita A . Role of the interaction of tumor necrosis factor-α and tumor necrosis factor receptors 1 and 2 in bone-related cells. Int J Mol Sci. (2022) 23:1481. doi: 10.3390/ijms23031481 35163403 PMC8835906

[26] Zhu X Zhang J Ge C Yu Y Wang P Yuan TF . Advances in cancer pain from bone metastasis. Drug Des Devel Ther. (2015) 9:4239–45. doi: 10.2147/DDDT.S87568 PMC4547660 26316696

[27] Taves S Berta T Chen G Ji RR. Microglia and spinal cord synaptic plasticity in persistent pain. Neural Plast. (2013) 2013:753656. doi: 10.1155/2013/753656 24024042 PMC3759269

[28] Tobinick EL. Targeted etanercept for treatment-refractory pain due to bone metastasis: two case reports. Clin Ther. (2003) 25:2279–88. doi: 10.1016/s0149-2918(03)80219-9 14512134

[29] Drynda A Drynda S Lohmann CH Bertrand J Kekow J. IL-17 regulates expression of cytokines in human osteoblasts rather than bone-specific genes. Osteologie. (2021) 30:49–56. doi: 10.1055/a-1177-5073

[30] Liu H Lv X Zhao X Yi L Lv N Xu W . Spinal astrocyte-derived interleukin-17A promotes pain hypersensitivity in bone cancer mice. Acta Pharm Sin B. (2024) 14:5249–66. doi: 10.1016/j.apsb.2024.09.016 39807339 PMC11725171

[31] Fontana F Giannitti G Marchesi S Limonta P. The PI3K/akt pathway and glucose metabolism: a dangerous liaison in cancer. Int J Biol Sci. (2024) 20:3113–25. doi: 10.7150/ijbs.89942 38904014 PMC11186371

[32] Pezet S Marchand F D’Mello R Grist J Clark A Malcangio M . Phosphatidylinositol 3-kinase is a key mediator of central sensitization in painful inflammatory conditions. J Neurosci. (2008) 28:4261–70. doi: 10.1523/JNEUROSCI.5392-07.2008 18417706 PMC2935680

[33] Wang L Yao M Yang J Peng J Peng Y Li C . Cancer-induced bone pain sequentially activates the ERK/MAPK pathway in different cell types in the rat spinal cord. Mol Pain. (2011) 7:48. doi: 10.1186/1744-8069-7-48 21722369 PMC3150304

[34] Calapai F Mondello E Mannucci C Sorbara EE Gangemi S Quattrone D . Pain biomarkers in cancer: an overview. Curr Pharm Des. (2021) 27:293–304. doi: 10.2174/1381612826666201102103520 33138755

[35] Spallone V Morganti R D’Amato C Greco C Cacciotti L Marfia GA. Validation of DN4 as a screening tool for neuropathic pain in painful diabetic polyneuropathy. Diabet Med J Br Diabet Assoc. (2012) 29:578–85. doi: 10.1111/j.1464-5491.2011.03500.x 22023377

[36] Zinboonyahgoon N Luansritisakul C. Neuropathic pain feature in cancer-induced bone pain: does it matter? A prospective observational study. Korean J Pain. (2023) 36:253–67. doi: 10.3344/kjp.22392 36973971 PMC10043784

[37] Thureau S Marchesi V Vieillard MH Perrier L Lisbona A Leheurteur M . Efficacy of extracranial stereotactic body radiation therapy (SBRT) added to standard treatment in patients with solid tumors (breast, prostate and non-small cell lung cancer) with up to 3 bone-only metastases: study protocol for a randomised phase III trial (STEREO-OS). BMC Cancer. (2021) 21:117. doi: 10.1186/s12885-021-07828-2 33541288 PMC7863429

[38] Schaub SK Tseng YD Chang EL Sahgal A Saigal R Hofstetter CP . Strategies to mitigate toxicities from stereotactic body radiation therapy for spine metastases. Neurosurgery. (2019) 85:729–40. doi: 10.1093/neuros/nyz213 31264703

